# Polyethylene Glycol‐Like Brush Polymer Conjugate of a Protein Drug Does Not Induce an Antipolymer Immune Response and Has Enhanced Pharmacokinetics than Its Polyethylene Glycol Counterpart

**DOI:** 10.1002/advs.202103672

**Published:** 2022-02-08

**Authors:** Imran Ozer, Garrett Kelly, Renpeng Gu, Xinghai Li, Nikita Zakharov, Parul Sirohi, Smita K. Nair, Joel H. Collier, Michael S. Hershfield, Angus M. Hucknall, Ashutosh Chilkoti

**Affiliations:** ^1^ Department of Biomedical Engineering Duke University Durham NC 27708 USA; ^2^ Department of Surgery Duke University School of Medicine Durham NC 27710 USA; ^3^ Department of Medicine Division of Rheumatology Duke University Medical Center Durham NC 27710 USA; ^4^ Department of Biochemistry Duke University School of Medicine Durham NC 27710 USA

**Keywords:** antigenicity, immunogenicity, pegloticase, pharmacokinetics, polyethylene glycol, polyethylene glycol antibody, uricase

## Abstract

Protein therapeutics, except for antibodies, have a short plasma half‐life and poor stability in circulation. Covalent coupling of polyethylene glycol (PEG) to protein drugs addresses this limitation. However, unlike previously thought, PEG is immunogenic. In addition to induced PEG antibodies, ≈70% of the US population has pre‐existing anti‐PEG antibodies. Both induced and preexisting anti‐PEG antibodies result in accelerated drug clearance, reduced clinical efficacy, and severe hypersensitivity reactions that have limited the clinical utility of uricase, an enzyme drug for treatment for refractory gout that is decorated with a PEG corona. Here, the authors synthesize a poly(oligo(ethylene glycol) methyl ether methacrylate) (POEGMA) conjugate of uricase that decorates the protein with multiple polymer chains to create a corona to solve these problems. The resulting uricase‐POEGMA is well‐defined, has high bioactivity, and outperforms its PEG counterparts in its pharmacokinetics (PK). Furthermore, the conjugate does not induce anti‐POEGMA antibodies and is not recognized by anti‐PEG antibodies. These findings suggest that POEGMA conjugation may provide a solution to the immunogenicity and antigenicity limitations of PEG while improving upon its PK benefits. These results transcend uricase and can be applied to other PEGylated therapeutics and the broader class of biologics with suboptimal PK.

## Graphical abstract

Here, the authors solve PEGylated uricase's polyethylene glycol (PEG) immunogenicity limitation by reformulating it using a nextgeneration stealth polymer poly(oligo(ethylene glycol) methyl ether methacrylate) (POEGMA). The uricase‐POEGMA conjugate has longer pharmacokinetics than the PEGylated drug, does not induce anti‐POEGMA antibodies, and is nonreactive to anti‐PEG antibodies. These results apply to most biologics with suboptimal pharmacokinetics and PEG immunogenicity and antigenicity.

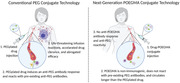

## 1 Introduction

Protein therapeutics are an important class of drugs due to their high potency and specificity.^[^ ^1^ ^]^ The protein‐drug market has grown steadily over the last decade, with an anticipated market size of $228.4 billion by the end of 2021.^[^ ^2^ ^]^ Despite their significant therapeutic promise, protein therapeutics, except for antibodies,^[^ ^3^ ^]^ are limited in the clinic due to their short half‐life and poor stability in circulation,^[^ ^4^ ^]^ necessitating frequent injections that result in peak‐and‐valley pharmacokinetics (PK), that aggravate treatment associated side‐effects and cost.^[^ ^5^ ^]^

Covalent conjugation of protein drugs to polyethylene glycol (PEG)—termed PEGylation—is a commonly used technology to overcome these delivery challenges.^[^ ^6^ ^]^ PEGylation improves the solubility and stability of protein drugs in circulation. It also extends their half‐life by increasing their hydrodynamic size (*R*_h_), thereby reducing their renal clearance and shielding immunogenic epitopes on the protein, thereby preventing protein drugs from clearance by the reticuloendothelial system.^[^ ^7^ ^]^ Despite the clear PK advantages conferred by PEGylation of biologics, it has significant shortcomings. First, PEG induces a significant anti‐PEG antibody response upon treatment with PEGylated therapeutics—defined as PEG immunogenicity.^[^ ^8^ ^]^ Analyses of the induced anti‐PEG response have revealed that both long EG repeating units and the hydrophobic end‐group of PEG act as immunogenic epitopes.^[^ ^9^ ^]^ In addition to these structural features, the inherent immunogenicity of the drug carrier also affects the anti‐PEG immune response.^[^ ^9^, ^10^ ^]^ The titer and affinity of PEG antibodies increase with an increasing number of immunogenic epitopes in the drug and increasing amount and density of PEG presented on the surface of the protein.^[^ ^11^ ^]^ Second, PEGylated therapeutics also bind to pre‐existing PEG antibodies—defined as PEG antigenicity—present in ≈70% of the US population at varying titers, possibly due to the widespread use of PEG in consumer products and therapeutics as an excipient.^[^ ^12^ ^]^ Together, the high titer of induced and pre‐existing PEG antibodies can abrogate the clinical efficacy of PEGylated drugs and result in life‐threatening hypersensitivity reactions,^[^ ^8^, ^13^ ^]^ which have led to the termination of clinical trials of several PEGylated drug candidates and the withdrawal of US Food and Drug Administration (FDA)‐approved PEGylated drugs from the market.^[^ ^13^, ^14^ ^]^

There are two classes of PEG conjugates: site‐specific and universal. In site‐specific PEG conjugates, a single PEG chain is typically attached via its chain end to a specific solvent‐accessible site on a protein; these conjugates typically have a protein: PEG stoichiometry of 1:1. Examples of FDA‐approved site‐specific conjugates are pegfilgrastim (Neulasta; Amgen), certolizumab pegol (Cimzia; UCB), and peginesatide (Omontys; Affymax and Takeda).^[^ ^15^ ^]^ In contrast, universal conjugates are an orthogonal class, where a large number of PEG chains are conjugated to the protein to create a PEG corona on the protein. Universal conjugates are typically synthesized when the protein drug is itself highly immunogenic so that the PEG corona masks immunogenic epitopes on the protein. This class of conjugates provides a stringent test of the intrinsic immunogenicity of new polymers, as a highly immunogenic protein conjugated to the polymer can stimulate antibody response against the conjugated polymer. Indeed, these adverse clinical implications of PEG immunogenicity were starkly illustrated with a PEGylated protein drug—pegloticase (Krystexxa; Horizon Pharma). Pegloticase is an FDA‐approved PEGylated porcine‐baboon chimeric uricase that is used to treat chronic refractory gout, a purine catabolism disease characterized by hyperuricemia and uric acid accumulation in the joints, leading to arthritis.^[^ ^8b^ ^]^ Unmodified uricase is limited as a drug because of its high immunogenicity due to the lack of endogenous uricase expression in higher primates^[^ ^16^ ^]^ and its low solubility at physiological pH, resulting in fast clearance from blood circulation, which limits its efficacy.^[^ ^11^, ^14^ ^]^ Even though PEGylated uricase solved these problems and was remarkably effective in 49% of gout patients who had failed all other treatments,^[^ ^8^, ^17^ ^]^ it still had significant problems. The uricase‐PEG conjugate induced a significant anti‐drug antibody (ADA) response that was primarily directed toward the PEG portion of the conjugate in 41% of patients, resulting in the accelerated clearance of subsequently administered doses of the drug.^[^ ^18^ ^]^ Moreover, ≈50% of patients with high titers of PEG antibodies experienced infusion reactions—26% characterized as severe and 6.5% as life‐threatening anaphylaxis—upon administration of subsequent doses of the drug due to activation of the complement system by the high titers of induced PEG antibodies, which prevented repeated administration of the drug necessary to eliminate tissue deposits of monosodium urate crystals.^[^ ^14a^ ^]^ The high titer and affinity of the anti‐PEG antibody response mounted toward pegloticase and its negative side‐effects were attributed to the immunogenicity of uricase.^[^ ^8^, ^19^ ^]^ Although concomitant immunosuppression with co‐administered rapamycin mitigates the induction of ADAs toward uricase‐PEG conjugates,^[^ ^20^ ^]^ this approach does not solve the root problem of PEG immunogenicity and may not apply to all PEGylated therapeutics limited with these problems. Hence, there is a critical unmet need to solve the problem caused by the PEG immunogenicity that has hampered the clinical utility of pegloticase and other PEGylated therapeutics.^[^ ^14a^ ^]^

To address these limitations of PEG, we have developed a next‐generation PEG‐like stealth polymer poly(oligoethylene glycol) methyl ether methacrylate (POEGMA). POEGMA is a brush polymer that breaks up the long and repetitive immunogenic ethylene glycol sequence in PEG into shorter oligoethylene glycol oligomers (EGX, where X denotes the number of EG repeats) and appends them as side‐chains on a poly(methyl methacrylate) backbone. We have previously shown that site‐specific and stoichiometric (1:1) POEGMA conjugates of a peptide drug improved upon the favorable PK/PD benefits of PEG by providing up to 6 days of in vivo efficacy after a single subcutaneous (s.c.) injection in mice.^[^ ^21^ ^]^ We have also demonstrated that site‐specific and stoichiometric peptide^[^ ^21b^ ^]^ and oligonucleotide^[^ ^22^ ^]^ conjugates of POEGMA did not show reactivity to PEG antibodies present in murine and human plasma, presumably because their short EG3 side‐chains lack the immunogenic epitope recognized by PEG antibodies. Finally, we have shown that site‐specific and stoichiometric conjugates of POEGMA with an RNA aptamer,^[^ ^22^ ^]^ a mildly immunogenic peptide, and a highly immunogenic protein—ovalbumin (OVA)^[^ ^21b^ ^]^—did not induce an anti‐POEGMA antibody response in mice even when administered with an adjuvant. These studies, however, were restricted to site‐specific conjugates, where the density of POEGMA is limited by the 1:1 stoichiometry of the conjugate, the relatively low thymus‐dependent immunogenicity of the peptide drug, and the low POEGMA dose used for in vivo efficacy studies.

An outstanding question, and one that is addressed in this paper, is how does a universal POEGMA conjugate of a highly immunogenic protein drug—uricase—with a high POEGMA stoichiometry (1:≈30) compare with its PEG counterparts in eliciting an antibody response? We posed this question as a stringent test of the intrinsic immunogenicity of this polymer, as the high immunogenicity of uricase has the potential to evoke a strong thymus‐dependent antibody response, similar to the immunogenic response elicited against PEG in the PEG conjugate of uricase.^[^ ^8^, ^17^, ^23^ ^]^ A secondary goal was to confirm that a universal POEGMA conjugate of a protein also confers the same PK benefits as a site‐specific POEGMA conjugate,^[^ ^21^, ^22^ ^]^ and remains unreactive—nonantigenic—to pre‐existing PEG antibodies.

We demonstrate that a universal POEGMA conjugate of uricase (uricase‐POEGMA) had a more favorable PK profile than PEGylated uricase. In addition, we show that uricase‐POEGMA is not recognized by PEG antibodies. Finally, and most importantly, we show that uricase‐POEGMA does not induce anti‐POEGMA antibodies even when conjugated to a highly immunogenic protein. These findings suggest a way to solve the problems limiting the clinical utility of uricase and other highly immunogenic proteins that require a PEG corona to mask their immunogenicity but that—ironically—induce an adverse antibody response to the conjugated PEG.

## 2 Results

### 2.1 Aggregate‐Free Uricase Expression with High Yield and Pharmacological Activity

**Figure 1:**
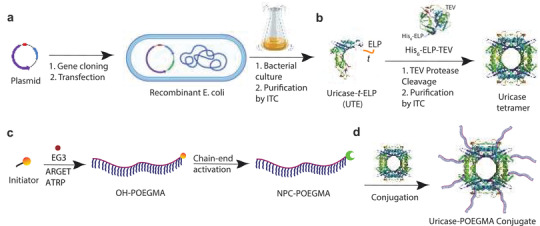
Overview of uricase‐POEGMA conjugate synthesis. a) Recombinant expression and purification of UTE. b) TEV protease‐mediated cleavage and purification by ITC to generate uricase tetramers. c) ARGET‐ATRP of OH‐functional POEGMA using an EG3 monomer, followed by activation of chain‐ends to NPC. d) Conjugation of NPC‐POEGMA to uricase tetramer, yielding uricase‐POEGMA. TEV protease and uricase images were from RCSB PDB with IDs of IQ31^[^ ^27^ ^]^ and 5FRC,^[^ ^28^ ^]^ respectively.

Uricase is a tetrameric protein comprised of four identical monomers.^[^ ^24^ ^]^ We developed a protocol to express uricase with high yield and stability and to purify it without the need for labor‐intensive chromatography methods by fusing uricase to a stimuli‐responsive elastin‐like polypeptide (ELP) to stabilize it during recombinant expression in *Escherichia coli* (*E. coli*).^[^ ^25^ ^]^ We fused an ELP to the C‐terminus of the uricase monomer at the gene level (**Figure** **1**a). We also inserted a Tobacco Etch Virus (TEV) protease recognition sequence—ENLYFQS; termed *t*—between the ELP and uricase monomer so that cleavage by TEV protease liberates the uricase monomers from the ELP after bacterial expression. The resulting fusion protein—termed uricase‐*t*‐ELP (UTE)—was recombinantly expressed in *E. coli* with a yield of ≈200 mg L^−1^ of shaker flask culture. We then exploited the stimuli‐responsive phase behavior of UTE that is imparted to the fusion protein by the ELP tag for chromatography‐free purification of UTE from the bacterial cell lysate by inverse transition cycling (ITC),^[^ ^26^ ^]^ yielding pure UTE, as assessed by sodium dodecyl sulfate‐polyacrylamide gel electrophoresis (SDS‐PAGE) (Figure S1, Supporting Information).

**Figure 2:**
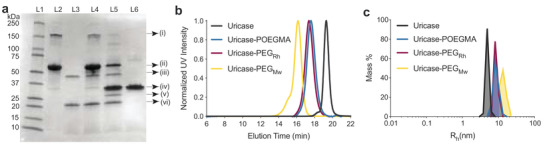
Synthesis and characterization of the uricase conjugates. a) SDS‐PAGE of TEV‐protease mediated UTE cleavage. L1: Molecular weight ladder; L2: i) undissociated UTE trimer, possibly due to ELP preventing complete disassociation and ii) pure UTE monomer; L3: iii) His_6_‐ELP‐TEV and vi) its truncation product His_6_‐ELP; L4: Reaction mix at *t* = 0; L5: Reaction mix *t* = 20 h showing iv) free uricase monomers and v) cleaved ELP; L6: iv) ITC‐purified uricase monomer. b) SEC traces of uricase variants. Peaks eluting at 18–19 and 19–21 min correspond to native octameric (0.8 mass%) and tetrameric (99.2 mass%) uricase, respectively, measured by multi‐angle light scattering. c) DLS analysis of uricase variants.

TEV protease was also recombinantly expressed in *E. coli* as a C‐terminus ELP fusion with an N‐terminus histidine (His) tag—termed His_6_‐ELP‐TEV.^[^ ^29^ ^]^ UTE was incubated with His_6_‐ELP‐TEV, which cleaved UTE between the Q and S in—ENLYFQS—the TEV protease recognition sequence and liberated uricase‐ENLYFQ from the ELP with ≈72% yield, allowing uricase tetramers to form spontaneously (Figures 1b and **2**a). Tetrameric uricase was then conveniently purified from His_6_‐ELP‐TEV, unreacted UTE, and cleaved ELP by a single round of ITC, as it was the only moiety that did not carry an ELP, yielding pure uricase tetramers (Figure 2a).

The uricase tetramer had a number‐average molecular weight (*M*_n_) of 136.6 kDa, as measured by size‐exclusion chromatography‐multi‐angle light scattering (SEC‐MALS) (Figure 2b and **Table** **1**). Uricase had a hydrodynamic size (*R*_h_) of ≈4.7 nm (Figure 2c and Table 1), as measured by dynamic light scattering (DLS), and comprised of 91.2% tetramer and 0.8 mass % octamer, which is a less‐frequent native conformation of uricase (Figure S2, Supporting Information).^[^ ^30^ ^]^ It was free of aggregates, indicated by the lack of larger molecules, as confirmed by SEC‐MALS and DLS.

**Table 1:** Summary of the conjugate characterization. The *M*_n_, *M*_w_, and *Ð* were measured by SEC‐MALS (data in Figure 2b). Conjugation stoichiometry was calculated by SEC‐MALS (*n* = 3). The *R*_h_ was measured by DLS shown in Figure 2c (*n* = 10). The enzymatic activity was determined by a biochemical assay (*n* = 5) that measures the ability of uricase to convert uric acid into allantoin. Data are reported as mean ± standard deviation. Not applicable (N/A)

Compound	*M*_n_ [kDa]	*M*_w_ [kDa]	*Ð*	Conjugation Stoichiometry [Polymer: Uricase]	*R*_h_ [nm]	Activity [U mg^−1^ uricase]
Uricase	136.6	136.6	1.00	N/A	4.7 ± 0.2	11.6 ± 1.3
Uricase‐POEGMA	417.5	426.1	1.09	27.0 ± 1.4	8.6 ± 1.5	12.1 ± 0.5
Uricase‐PEG_Rh_	228.3	252.7	1.10	8.9 ± 1.1	8.9 ± 0.3	10.8 ± 0.2
Uricase‐PEG_Mw_	440.6	443.6	1.11	29.3 ± 1.7	14.6 ± 1.5	11.4 ± 0.2

Uricase's pharmacological activity was quantified using a biochemical assay, where it catalyzed the conversion of its natural substrate—uric acid—to highly soluble allantoin, while producing hydrogen peroxide and carbon dioxide as byproducts.^[^ ^31^ ^]^ Our expression and purification protocol yielded uricase with an activity of ≈12 U mg^−1^ (Table 1), which is significantly higher than the activity of uricase from a commercial supplier (Sigma) that had an activity of only 4.4 U mg^−1^. In addition, uricase was stable for up to one week at 4 °C in phosphate‐buffered saline (PBS), indicated by the fact that >98 mass% of the molecules retained their original size (Figure S3, Supporting Information) and activity (data not shown).

### 2.2 Synthesis and Characterization of Well‐Defined Uricase‐POEGMA Conjugates

We synthesized POEGMA using a hydroxyl‐functional polymerization initiator (Figures S4 and S5, Supporting Information) and with a three‐ethylene glycol‐long (EG3) side‐chain, using activators regenerated by electron transfer atom transfer radical polymerization (ARGET‐ATRP),^[^ ^32^ ^]^ yielding hydroxyl‐functional POEGMA (OH‐POEGMA). The EG3 side‐chain length was chosen because longer side‐chains show binding to PEG antibodies,^[^ ^21^, ^33^ ^]^ while shorter side‐chains trigger phase transition into an insoluble phase at body temperature,^[^ ^34^ ^]^ prohibiting parenteral delivery.^[^ ^21b^ ^]^ The side‐chain length of OH‐POEGMA was confirmed by nuclear magnetic resonance (NMR) spectroscopy (Figure S6, Supporting Information). The OH‐POEGMA was monodisperse, with a low polydispersity (*Ð*) of <1.2 and had a weight‐average molecular weight (*M*_w_) of ≈10 kDa (Figure S7 and Table S1, Supporting Information), as analyzed by gel permeation chromatography‐MALS (GPC‐MALS). This *M*_w_ was chosen to match pegloticase's structure, in which a 10 kDa methoxy chain‐end functional PEG was conjugated to uricase.^[^ ^23b^ ^]^

We synthesized POEGMA conjugates of uricase by coupling nitrophenyl carbonate (NPC) functional POEGMA (NPC‐POEGMA) to the amine residues on solvent‐exposed lysine residues in a non‐site‐specific and nonstoichiometric manner, resulting in a physiologically stable urethane bond. We synthesized NPC‐POEGMA by activating the hydroxyl end group of OH‐POEGMA to NPC.^[^ ^35^ ^]^ The NPC chain activation was confirmed by NMR spectroscopy (Figure S8, Supporting Information) and did not alter POEGMA's *M*_n_, *M*_w_, and *Ð* (Table S1, Supporting Information).

The resulting conjugate had an average of ≈27 POEGMA chains per uricase tetramer (Figure 2b and Table 1). The conjugation stoichiometry was chosen to match pegloticase's structure, comprising ≈32 PEG chains on average per uricase tetramer^[^ ^23b^ ^]^ that shield uricase's immunogenic epitopes that are distributed over its surface and that improve the drug's PK. We also synthesized a uricase‐PEG conjugate with matching conjugation stoichiometry and *M*_w_—termed uricase‐PEG_Mw_—to use as a control. The conjugates were aggregate‐free and monodisperse with a low *Ð* of <1.2 and had near‐identical conjugation stoichiometries (Figure 2b and Table 1). Despite their identical conjugation stoichiometry, the conjugates significantly differed in their *R*_h_ (Figure 2c and Table 1) due to POEGMA's more compact brush architecture than linear PEG (Table S1, Supporting Information).^[^ ^36^ ^]^ Given that the renal clearance rate may be affected by the *R*_h_ of the conjugates and complicate head‐to‐head comparison of PK,^[^ ^37^ ^]^ we also synthesized an *R*_h_‐matched uricase‐PEG conjugate—termed uricase‐PEG_Rh_. The uricase‐PEG_Rh_ conjugate was monodisperse and had a PEG conjugation stoichiometry of ≈9 per tetramer (Figure 2c and Table 1).

The conjugates did not differ in enzymatic activity and were as effective as uricase, indicating that PEG or POEGMA conjugation did not affect the pharmacological activity of uricase (Table 1). In contrast, PEG^[^ ^38^ ^]^ and POEGMA^[^ ^21a^ ^]^ conjugation to peptide or protein drugs can result in significant loss of activity compared to the unmodified drug when the target of the drug is a large biological moiety, such as a membrane‐bound receptor.^[^ ^21a^ ^]^ We attributed this result, first, to the small size of uric acid as a substrate, which should easily penetrate through the corona of PEG and POEGMA chains to reach uricase. A POEGMA‐conjugate of asparaginase also showed only a minimal (threefold) change in activity—catalysis of a small molecule substrate asparagine—supporting our findings.^[^ ^39^ ^]^ This result also suggests that the reaction conditions used to conjugate POEGMA or PEG to uricase did not cause denaturation of the enzyme.

### 2.3 Pharmacokinetics

We next investigated the PK of uricase‐POEGMA and compared it with unmodified uricase, uricase‐PEG_Mw_, and uricase‐PEG_Rh_. The uricase variants were fluorescently labeled to track them in vivo (Figure S9, Supporting Information). We first confirmed that uricase‐POEGMA would not phase transition upon in vivo administration, as POEGMA undergoes lower critical solution temperature phase transition behavior.^[^ ^40^ ^]^ The cloud point temperature (*T*_t_) of uricase‐POEGMA was well above body temperature (≈42 °C) at the injection concentration of 0.6 mg (uricase‐based) mL^−1^ in PBS (Figure S10, Supporting Information). Next, the uricase variants were intravenously (i.v.) administered into 6‐week‐old male C57BL/6J mice (*n* = 5), and the plasma drug concentration was tracked over time, post injection. These data were then analyzed to determine the PK parameters.

**Figure 3:**
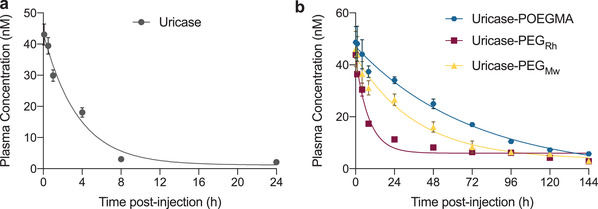
PK of the uricase variants. Plasma concentrations of sterile, endotoxin‐free, and fluorescently labeled a) unmodified uricase and b) uricase conjugates in C57BL/6J (*n* = 5) mice after a single i.v. administration at a dose of 36.6 nmol kg^−1^. Plasma concentration of the drugs was tracked by collecting blood at predetermined time points for 144 h, followed by processing them into plasma and measuring their fluorescence. Data show the mean ± standard error of the mean (SEM). Data were fit to a one‐phase exponential decay curve.

The treatments resulted in near‐constant initial plasma concentration (*C*_o_) of the two PEG conjugates, the POEGMA conjugate, and unmodified uricase, indicating successful equimolar drug administration (**Table** **2**). Unmodified uricase had a short elimination half‐life (*t*_1/2_ _elimination_), consistent with previous results (**Figure** **3**a and Table 2).^[^ ^31^ ^]^ All conjugates had a longer *t*_1/2_ _elimination_ than unmodified uricase, indicating that both PEG and POEGMA successfully extended uricase's PK (Figure 3b and Table 2). Uricase‐PEG_Rh_ showed biphasic PK, indicated by faster clearance upon administration, followed by a slower clearance of the remaining drug. We speculate that this could be due to its precipitation in circulation, given that it has the lowest conjugation stoichiometry among the conjugates that could potentially negatively impact its stability. The PEG conjugates significantly differed in *t*_1/2_ _elimination_. Uricase‐PEG_Mw_ prolonged uricase's circulation by ≈16‐fold compared to uricase (*t*_1/2_ _elimination_ of 35.5 h vs 2.2 h), while uricase‐PEG_Rh_ had only an approximately sixfold longer circulation than uricase (*t*_1/2_ _elimination_ of 13.1 h vs 2.2 h). Uricase‐PEG_Rh_ had much lower plasma exposure than uricase‐PEG_Mw_, indicated by the lower area under the curve (AUC) (Table 2). In addition to the lower stability of uricase‐PEG_Rh_, this PK difference could also be attributed to the larger size of uricase‐PEG_Mw_, allowing it to overcome renal clearance more efficiently. These findings are consistent with previous studies, where improved PK was observed with increased *R*_h_^[^ ^37^ ^]^ and PEG conjugation stoichiometry.^[^ ^31^, ^41^ ^]^

**Table 2:** Summary of PK parameters. PK parameters were determined from the data in Figure 3 using noncompartmental analysis. Data shows the mean ± SEM and analyzed by two‐way repeated‐measures ANOVA, followed by post‐hoc Tukey's multiple comparison test. *Derived from a curve‐fit of the data in Figure 3

Mice	Treatment	*C*_o_ [nm]*	*t*_½ elimination_ [h]	AUC [nm × h]
Naïve Mice (1st injection)	Uricase	47.3 ± 1.7	2.2 ± 0.1	190 ± 16
	Uricase‐POEGMA	47.4 ± 2.0	46.2 ± 1.3	2825 ± 121
	Uricase‐PEG_Rh_	37.1 ± 0.4	13.1 ± 0.5	1231 ± 80
	Uricase‐PEG_Mw_	41.3 ± 1.9	35.5 ± 1.0	1991 ± 153
Immunized Mice (5th injection)	Uricase	43.0 ± 0.5	2.9 ± 0.2	219 ± 29
	Uricase‐POEGMA	47.5 ± 1.6	49.6 ± 2.0	3064 ± 193
	Uricase‐PEG_Mw_	34.1 ± 1.1	29.6 ± 0.1	1623 ± 160

Notably, uricase‐POEGMA outperformed both uricase‐PEG_Rh_ and uricase‐PEG_Mw_ by extending uricase's circulation by ≈21‐fold compared to uricase (*t*_1/2_ _elimination_ of 46.1 h vs 2.2 h). Together, these results indicated that uricase‐POEGMA has a more favorable PK than the PEG conjugates. We do not believe that the superior PK profile of uricase‐POEGMA is solely due to kidney clearance rates as the uricase‐POEGMA has better PK as seen by its larger AUC than its size‐matched PEG control, uricase‐PEG_Rh_. We previously attributed the superior PK profile of s.c. injected exendin‐POEGMA conjugates to POEGMA's amphiphilic structure, allowing slower absorption from the s.c. compartment than PEG conjugates, which delayed elimination of the conjugate.^[^ ^21b^ ^]^ This rationale however does not apply to the uricase conjugates, given their i.v. administration directly into the bloodstream that bypasses the absorption phase of the drug. We speculate that the superior PK profile of uricase‐POEGMA is related to POEGMA's brush architecture, consistent with previous studies that have shown that the architecture of stealth polymers affects their PK profiles,^[^ ^37^, ^42^ ^]^ though the precise molecular details remain to be elucidated.

### 2.4 Immunogenicity

Having shown the PK benefits of POEGMA conjugation to uricase, we next investigated its immunogenicity. This experiment was motivated by the high titers of anti‐PEG antibodies induced in response to pegloticase treatment that had led to the accelerated clearance of the subsequent doses of the drug and infusion reactions.^[^ ^8^, ^11^, ^17^, ^23^ ^]^ The PEG‐specific immune response was due to the high immunogenicity of the drug that is a nonhuman porcine‐baboon chimeric uricase, that induces a thymus‐dependent immune response, where uricase‐PEG is taken up by dendritic cells and uricase‐derived peptides are presented by MHC class II molecules on the dendritic cells to activate follicular helper T cells that provide help to induce affinity maturation and class switching of anti‐PEG B cells.^[^ ^10^, ^43^ ^]^ We hypothesized that uricase‐POEGMA conjugates would not induce anti‐POEGMA antibodies and solve the problem caused by PEG immunogenicity of uricase‐PEG conjugates. This hypothesis was based on our previous findings, where a site‐specific and stoichiometric (1:1) POEGMA conjugate of a mildly immunogenic peptide^[^ ^21b^ ^]^ drug did not induce any anti‐POEGMA antibodies. We attributed the lack of POEGMA immunogenicity to its short EG side chains and mild thymus‐dependent immunogenicity of the peptide. However, a conjugate of a highly immunogenic protein—uricase—with high a POEGMA density (≈27 POEGMA vs 1 per drug) was of great interest, as it poses the most stringent test in mice of the intrinsic immunogenicity of POEGMA to date, and data that is critical for the development of POEGMA conjugates of biologics as drug candidate.

**Figure 4:**
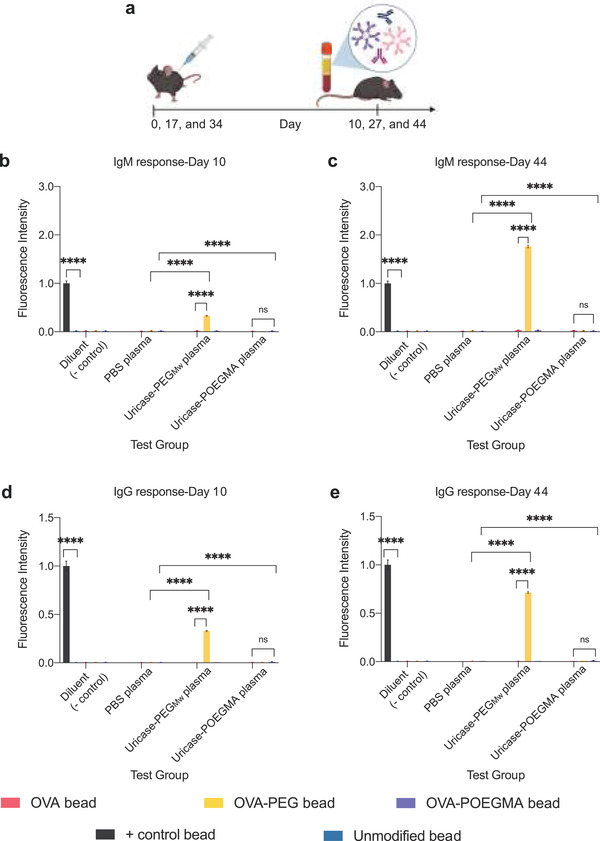
ADA response induced by uricase‐POEGMA and uricase‐PEG conjugates. a) Treatment and blood collection regimen (*n* = 10 mice per group). ADA response was measured for each mouse using a validated Luminex multiplexed assay (*n* = 6 per plasma sample). IgM response on b) Day 10 and c) Day 44. IgG response on d) Day 10 and e) Day 44. OVA‐PEG‐ and OVA‐POEGMA‐coupled beads show the PEG‐specific and POEGMA‐specific immune response in plasma samples of mice treated with PBS, uricase‐PEG_Mw_, and uricase‐POEGMA. Data were normalized to the signal measured from mouse IgG‐ and IgM‐coupled beads (positive controls) and are plotted as the average ADA response in a treatment group ± SEM. Data were analyzed by two‐way repeated‐measures ANOVA, followed by post‐hoc Tukey's multiple comparison test. A test was considered statistically significant when *p* < 0.05. ****p < 0.0001. Not significant (ns).

We tested this hypothesis by repeatedly administrating sterile and endotoxin‐free PBS, candida‐derived uricase‐PEG_Mw_, and uricase‐POEGMA into 6‐week‐old naïve C57BL/6J mice at a dose of 36 nmol kg^−1^, followed by blood collection and processing into plasma (see the dosing and blood collection regimen in **Figure** **4**a). We selected the s.c. injection route—rather than i.v.*—*because the conjugates are exposed to the lymphatic system during absorption into the blood from the s.c. compartment, revealing their immunogenic potential better.^[^ ^44^ ^]^ The temporal regimen of injection every 17 days was selected because it allowed us to eliminate the interference of residual circulating drug on subsequent immunoassays of the ADA response over time, as the drug is eliminated by day 6 (Figure 3).

We used a previously described validated Luminex multiplexed immunoassay.^[^ ^21b^ ^]^ Briefly, the assay uses drug‐coupled and fluorescently barcoded magnetic beads to assess the subtype and specificity of the ADA response (Figure S11, Supporting Information). Briefly, the specificity of the antibody response to POEGMA or PEG was tested using an unrelated protein—namely OVA—and its PEG and POEGMA conjugates for bead coupling, yielding OVA‐, OVA‐PEG‐, and OVA‐POEGMA‐coupled beads. If a signal was detected toward these beads in uricase‐PEG_Mw_‐ or uricase‐POEGMA‐treated mice plasma, it would indicate PEG‐ or POEGMA‐specific antibodies, respectively. The OVA‐coupled bead set was used as a control for cross‐reactivity between uricase and OVA. PBS‐treated mouse plasma was used as a negative control to assess effects of plasma. Mouse IgM‐ and IgG‐coupled beads incubated in the assay diluent were used as positive control beads for the assay, while the unmodified and drug‐coupled beads incubated in the assay diluent were used as negative control beads.

The positive control beads incubated in the assay diluent resulted in a high signal, while unmodified and drug‐coupled beads had minimal signal (Figure 4b). No significant background signal was detected, indicated by the lack of signal derived from the plasma samples of mice treated with PBS (Figure 4b–e). We confirmed the lack of PEG‐ or POEGMA‐ specific pre‐existing antibodies by testing pretreatment plasma samples (Figure S12, Supporting Information). The OVA‐coupled bead had a minimal signal in uricase‐PEG_Mw_‐ and uricase‐POEGMA‐treated mice plasma, indicating a lack of cross‐reactivity (Figure 4b–e). Uricase‐PEG_Mw_ induced PEG‐specific IgM response by Day 10, indicated by the significant ADA binding to OVA‐PEG‐coupled beads in uricase‐PEG_Mw_‐treated mice plasma (Figure 4b). The PEG‐specific ADA response persisted until Day 44 (Figure S13a, Supporting Information, and Figure 4c), increasing with the increasing number of injections (Figure S14a, Supporting Information). Uricase‐PEG_Mw_ also resulted in a robust PEG‐specific IgG response (Figure 4d,e and Figure S13b, Supporting Information), and the titer increased with each injection (Figure S14b, Supporting Information), indicating class‐switching. This strong PEG‐specific IgG response was due to the activation of the immune system by candida uricase‐derived peptides, allowing the immune system to mount a thymus‐dependent immune response, consistent with the literature.^[^ ^10b^ ^]^ Strikingly, uricase‐POEGMA induced no anti‐POEGMA IgM or IgG response (Figure 4b–e), indicated by the lack of signal detected by the OVA‐POEGMA bead. These findings corroborated with our previous findings and suggested that POEGMA may not induce an anti‐POEGMA response even when presented to the immune system at extremely high densities on an immunogenic protein. However, we acknowledge that these experiments do not conclusively establish the lack of immunogenicity of POEGMA as they were performed in C57BL/6J mice that induce a weaker antibody response to thymus‐dependent antigens than other mouse models, such as BALB/c.

### 2.5 The Effect of Induced Polyethylene Glycol Antibodies on Pharmacokinetics

We next investigated if induced PEG antibodies affected the PK of the circulating drug. We hypothesized that the PEG antibodies would result in early clearance of the uricase‐PEG_Mw_ upon repeated exposure, while no change would be observed in uricase‐POEGMA's PK due to the lack of a POEGMA‐specific immune response. We repeatedly administered uricase‐PEG_Mw_ and uricase‐POEGMA into mice to test this hypothesis, followed by tracking plasma drug concentrations and comparing PK data from the first and fifth injection. We also repeatedly administered a separate cohort of mice with uricase as a control for specificity. If no change in uricase PK were observed upon repeated exposure while uricase‐PEG_Mw_ showed accelerated clearance, it would indicate a PEG antibody‐mediated drug elimination.

The PK of the uricase and uricase‐POEGMA treatments did not change with the repeated injections (Table 2 and Figure S15a,b, Supporting Information), indicating that neither uricase nor POEGMA induced PK‐altering antibodies upon treatment, corroborating the ADA results. Uricase‐PEG_Mw_ had a significantly shorter *t*_1/2_ _elimination_ (35.5 vs 29.6 h) upon repeated exposure (Table 2 and Figure S15c, Supporting Information). We attributed this altered PK profile to the presence of PK‐altering PEG antibodies. These findings are consistent with the literature, where anti‐PEG antibodies abrogated the efficacy of subsequent pegloticase injections by altering the drug's PK.^[^ ^23b^ ^]^

### 2.6 Polyethylene Glycol Antigenicity

Having shown that PEG antibodies led to the early clearance of uricase‐PEG_Mw_, we next investigated if uricase‐POEGMA had any reactivity to PEG antibodies. We hypothesized that uricase‐POEGMA would not show antigenicity PEG antibodies based on our previous results, where we had shown that a site‐specific exendin‐POEGMA conjugate was not recognized by PEG antibodies in an in vitro immunoassay.^[^ ^21a^ ^]^ However, the much higher POEGMA stoichiometry of the uricase‐POEGMA conjugate than the site‐specific exendin‐POEGMA (≈27 POEGMA chains vs 1) justifies the need for further analysis of PEG antigenicity because the spatial distribution of POEGMA antigens on the uricase surface could alter epitope exposure to PEG antibodies, and thereby result in recognition of uricase‐POEGMA by PEG antibodies.

**Figure 5:**
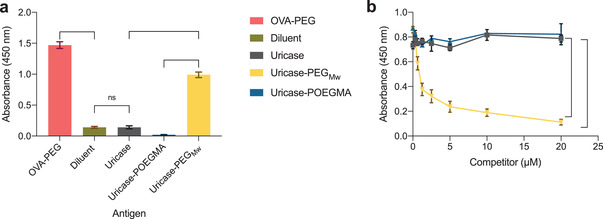
PEG antibodies do not cross‐react with uricase‐POEGMA. The reactivity of PEG antibodies to uricase, uricase‐PEG_Mw_, and uricase‐POEGMA was tested using a) indirect and b) competitive ELISA. In indirect ELISA, the binding of polyclonal PEG antibodies present in OVA‐PEG‐immunized mouse plasma to absorbed antigens is measured (*n* = 4). In the competitive ELISA, exendin‐PEG is absorbed onto the wells of 96‐well ELISA plates. The antigens (uricase, uricase‐PEG_Mw_, and uricase‐POEGMA) (*n* = 4) are premixed with OVA‐PEG‐treated mouse plasma and compete with the adsorbed exendin‐PEG for binding to the PEG antibodies present in the mouse plasma. Data represent the mean absorbance ± SEM and were analyzed using two‐way ANOVA, followed by post‐hoc Tukey's multiple comparison test. A test was considered statistically significant when *p* < 0.05. *p < 0.05, **p < 0.01, ***p < 0.001, and ****p < 0.0001. Not significant (ns).

We tested the PEG antigenicity of uricase, uricase‐PEG_Mw_, and uricase‐POEGMA using indirect (**Figure** **5**a) and competitive (Figure 5b) ELISA. In indirect ELISA, we absorbed conjugates or controls to the surface of wells of a 96‐well‐plate such that the wells had near‐equal amounts of adsorbed PEG/POEGMA and uricase, then incubated the wells with OVA‐PEG immunized mouse plasma available from another study that contains PEG antibodies.^[^ ^21b^ ^]^ Diluent and OVA‐PEG were used as negative and positive controls, respectively, for this assay. OVA‐PEG had a significant signal, while diluent resulted in only a minimal background (Figure 5a). Uricase‐PEG_Mw_ had a high signal, indicating that the conjugate reacted with the PEG antibodies present in the plasma sample. However, uricase had no significant absorbance because it lacks PEG. Notably, uricase‐POEGMA showed no reactivity to the PEG antibodies, suggesting that it was not antigenic to PEG antibodies even when presented at a high density on the surface of uricase.

The competitive ELISA confirmed the indirect ELISA results. The wells were coated with exendin‐PEG available from another study.^[^ ^21b^ ^]^ The antigens (uricase, uricase‐PEG_Mw_, and uricase‐POEGMA) (*n* = 4) were premixed with OVA‐PEG‐treated mouse plasma and competed with the adsorbed exendin‐PEG for binding to the PEG antibodies present in the mouse plasma. Consistent with the indirect ELISA results, uricase‐PEG_Mw_ successfully competed with exendin‐PEG, indicated by the significantly lower absorbance with increasing uricase‐PEG_Mw_ concentration (Figure 5b). However, uricase did not compete with exendin‐PEG for binding to PEG antibodies because it does not contain PEG. Notably, uricase‐POEGMA did not bind to PEG antibodies, confirming that PEG antigenicity is eliminated by a universal POEGMA conjugate with a high density of POEGMA on the surface of the protein.

## 3 Discussion

PEGylated uricase is used to treat refractory gout seen in over 10 million patients in the United States that has an increasing prevalence worldwide.^[^ ^45^ ^]^ Uricase reduces plasma uric acid levels within 24–72 h of administration and dissolves tophi—the urate deposits in the joints—effectively.^[^ ^8^, ^17^ ^]^ However, during the clinical trials of a PEGylated uricase—pegloticase, it was discovered that the conjugate rapidly induced anti‐PEG antibodies in ≈41% of patients.^[^ ^18^ ^]^ Pegloticase also showed reactivity to pre‐existing PEG antibodies. Even though pegloticase gained FDA approval in 2011 and has been dramatically effective in treating 49% of the chronic refractory gout patients who have failed all other therapies, PEG immunogenicity has limited its utility because anti‐PEG antibodies caused accelerated blood clearance and infusion reactions.^[^ ^8^, ^17^, ^23^ ^]^ In addition to the treatment of gout, a well‐tolerated and long‐acting uricase conjugate would also be useful for treating hyperuricemia that can occur following cancer chemotherapy—termed as tumor lysis syndrome—and organ transplantation, both of which can result in acute uric acid nephropathy.^[^ ^46^ ^]^ Rasburicase is a non‐PEGylated uricase that has been approved to manage plasma uric acid levels in patients with leukemia, lymphoma, and solid tumor malignancies who are receiving anticancer therapy that is expected to result in tumor lysis and subsequent elevation of plasma uric acid. However, rasburicase causes severe infusion reactions, hemolysis, and anaphylaxis in patients, limiting its use. Small molecule‐based uric acid reducers, such as allopurinol, probenecid, and febuxostat, are ineffective in treating refractory gout and have significant liver and kidney toxicity.^[^ ^47^ ^]^

We chose to conjugate POEGMA to uricase because it exemplifies the immunogenicity challenges in developing a universal—multi‐site—conjugate of highly immunogenic drugs. We conjugated POEGMA with an *M*_w_ of 10 kDa to the amine‐functional lysine residues on uricase in a non‐site‐specific and nonstoichiometric manner, yielding uricase‐POEGMA. We also synthesized *R*_h_‐ and *M*_w_‐matched uricase‐PEG conjugates as controls. The resulting uricase‐POEGMA conjugate had a uric acid conversion activity similar to unmodified uricase, indicating that POEGMA conjugation did not affect its pharmacological activity. Uricase‐POEGMA also had an *R*_h_ above the renal excretion threshold that endowed it with prolonged blood circulation. Notably, uricase‐POEGMA had a longer blood circulation half‐life and larger plasma AUC than the *M*_w_‐ and *R*_h_‐matched uricase‐PEG conjugates after a single i.v. administration. Together, these results show that POEGMA formulated uricase retains the biological activity of uricase‐PEG conjugates and improves the PK of the drug.

We also showed that POEGMA conjugation may suggest a way to solve the immunogenicity problem of PEGylated uricase, indicated by the lack of anti‐POEGMA antibodies upon repeated injections into C57BL6/J mice. In contrast, treatment with uricase‐PEG conjugate induced an intense and persistent anti‐PEG antibody response, which accelerated the clearance of the drug, similar to the results observed in the clinical trials of pegloticase. Notably, we observed a significant IgM and IgG response to the PEG component of the uricase‐PEG conjugate on day 10 after initial injection of the conjugate, which then strengthened by day 44 after administration of two more doses dose of the conjugate. In contrast, the anti‐POEGMA IgM and IgG response in mice treated to uricase‐POEGMA were minimal. These results are even more impressive than our previous findings on the lack of an anti‐POEGMA immune response, given that POEGMA remains non‐immune‐reactive even when presented to the immune system conjugated to a highly immunogenic thymus‐dependent antigen such as uricase. These results suggest that POEGMA is significantly less immunogenic than PEG, a feature that could be attributed to its short EG3 side chains. We acknowledge that these results are based on experiments conducted in C57BL/6J mice, which is known to elicit lower antibody responses as compared to the BALB/c mouse model.

Finally, we showed that uricase‐POEGMA is not reactive to pre‐induced anti‐PEG murine antibodies. The lack of reactivity toward anti‐PEG antibodies can be attributed to the short EG3 side chains of POEGMA that presumably lack the epitope, thereby preventing recognition by anti‐PEG antibodies. These results suggest that POEGMA can also address the growing problem of PEG antigenicity and can be safely used in an anti‐PEG antibody‐positive patient population. These findings are timely because ≈70% of the US population is anti‐PEG antibody positive. Like drug‐induced PEG antibodies, pre‐existing PEG antibodies also interfere with PEGylated drugs in individuals with high‐titers of anti‐PEG antibodies, rendering PEGylated drugs ineffective and inducing severe hypersensitivity reactions, as demonstrated by the early termination of a late‐stage clinical trial of a PEGylated drug due to severe PEG‐related hypersensitivity reactions and death.^[^ ^48^ ^]^ Given that billions of people worldwide are being administered PEG‐containing vaccines for acute respiratory syndrome coronavirus‐2 (SARS‐CoV‐2), the PEG antigenicity problem is anticipated to exponentially grow in the near future.

Importantly, our findings transcend uricase as a drug and apply to other PEGylated therapeutics that are facing significant PEG‐related challenges in the clinic or that were previously withdrawn from the market due to PEG‐induced severe infusion reactions and anaphylaxis, such as pegasparaginase (Oncospar; Ovation) and peginesatide (Omontys; Affymax and Takeda).^[^ ^14a^ ^]^ Pegasparaginase is PEGylated asparaginase used in the clinic to treat acute lymphoblastic leukemia. Asparaginase is short‐acting and highly immunogenic, resulting in neutralizing antibodies in 20–30% of patients and leading to the loss of efficacy upon repeated administrations. These problems necessitated PEG conjugation.^[^ ^39^ ^]^ Unfortunately, nearly half of the patients have developed anti‐PEG antibodies toward Pegasparaginase, resulting in accelerated blood clearance of the subsequently administered doses, loss of therapeutic efficacy, and severe hypersensitivity reactions.^[^ ^14a^ ^]^ Similarly, Peginesatide—a PEGylated recombinant erythropoietin‐stimulating agent used to treat anemia in patients with chronic kidney disease—was withdrawn from the market due to life‐threatening and fatal infusion reactions observed immediately after the first administration of the drug.^[^ ^11^, ^14^, ^49^ ^]^ Although no reports have been published on the mechanisms behind these infusion reactions and the role of PEG antibodies, the similarity of the clinical manifestations to that observed with pegloticase and other PEGylated therapeutics suggested their potential role.^[^ ^11^, ^14^ ^]^ Hence, there is a critical unmet need to solve the problem caused by the PEG immunogenicity and antigenicity, leading to life‐threatening and even fatal complications and limiting the utility of otherwise effective PEGylated drugs.

The POEGMA conjugate technology also applies to most biologics with a suboptimal PK and has significant advantages over other drug delivery methodologies. Other than PEGylation, the most widely applied methodology in the clinic to overcome these delivery challenges of biologics is to fuse them to Fc fragments, resulting in Fc fusions. Although Fc fusions confer significant PK benefits, their utility in drug delivery is limited by several factors. First, not all biologics can be fused to Fc fragments. Genetic fusion of a protein drug to an Fc fragment requires that the drug be fused to the N‐ or C‐terminus, limiting the site of fusion, and can negatively impact its expression level and lower the potency of the drug.^[^ ^50^ ^]^ Importantly, this strategy cannot be applied to highly immunogenic drugs like uricase and asparaginase, which require masking the immunogenic epitopes on the protein. We note that in principle, protein engineering can eliminate some of the immunogenic epitopes, thereby theoretically allowing the deimmunized protein to be grafted on an Fc scaffold. However, this approach is also limited by the number of epitopes that can be successfully deimmunized by site‐directed mutagenesis without deleteriously impacting the stability and activity of the protein. We also note that POEGMA is not the only alternative to PEG. Although they have not been approved for clinical use yet, other nonfouling polymers such as zwitterionic polymers are under development as an alternative to PEG.^[^ ^51^ ^]^

In summary, we have synthesized a well‐defined POEGMA conjugate of candida uricase with high uric acid conversion activity. The resulting uricase‐POEGMA suggests an approach to solve its PEG counterparts' immunogenicity and antigenicity limitations while improving upon their PK benefits with its brush architecture, suggesting that it can address the unmet clinical need for a well‐tolerated, effective, and long‐acting uricase for long‐term chronic refractory gout treatment. These findings transcend uricase conjugates and apply to salvage PEGylated therapeutics and the broader area of most biologics with short PK. Future studies include testing the immunogenicity of the conjugate in BALB/c mice that have higher antibody responses than C57BL/6J mice and pharmacodynamics of the conjugate in disease‐relevant animal models.

## 4 Experimental Section

### Gene Cloning

The gene sequence for *Candida utilis* uricase (Acc. No.: A0A1E4S4E2) was codon‐optimized for *E. coli* and cloned into a modified PET‐24a+ vector using restriction digest with AcuI and BseRI. A previously described seamless cloning method^[^ ^52^ ^]^ was used to clone in a C‐terminal TEV protease recognition site (ENLYFQ; *t*), followed by an ELP, consisting of 60 repeats of a VPGVG pentamer. The final construct encoding UTE was transformed into and expressed by BL21(DE3) *E. coli*. Next, an ELP‐tagged TEV protease was cloned as previously described.^[^ ^29^ ^]^ Briefly, the gene encoding for TEV protease (Acc. No.: Q0GDU8, residues 73‐302) was PCR‐amplified and cloned into PET‐24a+. A six‐residue N‐terminal His‐tag (His_6_) and an ELP consisting of 60 repeats of the VPGVG pentamer were seamlessly cloned onto the TEV protease gene to generate the His_6_‐ELP‐TEV construct. The resulting vector was transformed into BL21(DE3) *E. Coli*.

### Protein Expression and Purification

UTE‐expressing *E. coli* cultures were grown in shake flasks at 25 °C for 4 h in 2XYT media, induced with 0.5 mm isopropyl *β*‐D‐1‐thiogalactopyranoside (IPTG), and grown overnight at 16 °C. Cells were harvested by centrifugation at 3400 × *g* and 4 °C for 10 min and resuspended in cold borate buffer (100 mm sodium borate and 1 mm ethylenediaminetetraacetic acid [EDTA], pH 10). The cells were lysed by sonication (Q500 sonicator, QSonica, Newtown, CT) pulsed at 10 s on and 30 s off for a total sonication time of 180 s. DNA was precipitated by adding 10% polyethyleneimine, and lysates were clarified by centrifugation at 24 000 × *g* and 4 °C for 10 min. UTE was purified from the clarified lysate by ITC, as follows.^[^ ^26a^ ^]^ Briefly, the lysate was brought to room temperature, and the phase separation of UTE was triggered by the addition of 0.1 m ammonium sulfate. The insoluble coacervate, containing UTE, was pelleted by centrifugation at 24 000 × *g* and 35 °C for 10 min. The pellets were resuspended in cold borate buffer on a rotator at 4 °C for 1 h, and contaminants were removed by centrifugation at 24 000 × *g* and 4 °C for 10 min. This process was repeated for one or two cycles to reach greater than 95% purity of UTE. His_6_‐ELP‐TEV was expressed, harvested, lysed, and clarified as described above for UTE, followed by purification by nickel affinity chromatography and dialysis into Tris‐EDTA buffer. Purity was determined by SDS‐PAGE using gel densitometry analysis (Biorad). Protein concentration was determined by UV–vis spectroscopy using an ND‐1000 Nanodrop spectrometer (Thermo Scientific).

### Tobacco Etch Virus Protease Cleavage

UTE was cleaved by TEV protease to remove the ELP and produce free uricase as previously described with slight modifications.^[^ ^53^ ^]^ Briefly, the reaction mixture consisted of a 1:10 ratio of His_6_‐TEV‐ELP to UTE by absorbance at 280 nm in borate reaction buffer (100 mm sodium borate pH 9.2, 1 mm EDTA, and 10 mm dithiothreitol). The reaction was incubated on a rotator for 20 h at 4 °C. Finally, the His_6_‐ELP‐TEV, uncleaved UTE, and cleaved ELP were separated from uricase by adding 0.2 m ammonium sulfate to trigger phase separation of moieties with an ELP, followed by centrifugation at 24 000 × *g* and 25 °C for 10 min. The supernatant that contained the free tetrameric uricase was collected and dialyzed into a 50 mm carbonate buffer containing 100 mm NaCl pH 9.2 using a dialysis cassette with a molecular weight cut‐off (MWCO) of 10 kDa. The purity was determined by SDS‐PAGE using gel densitometry analysis (Biorad).

### Synthesis of ATRP Initiator

4‐amino‐1‐butanol (5 g, 0.0561 mol) and triethylamine (6.24 g, 0.0624 mol) were transferred into a reaction flask (flask 1) and dissolved in 20 mL dichloromethane (DCM). *α*‐bromoisobutyryl bromide (12.8 g, 0.0567 mol) was dissolved in 1 mL DCM in another flask (flask 2). The solution in flask 2 was drop‐wise transferred into flask 1 by a syringe under an inert atmosphere. The reaction mixture was stirred for 16 h at room temperature. The resulting mixture was filtered, and the organic phase was collected. Next, 20 mL of potassium hydroxide (5% v/v) was added and stirred for 2 h. The organic phase was separated using a separatory funnel and washed with 1 n NaOH, 1 n HCl, and saturated NaCl, respectively. The organic phase was dried over anhydrous MgSO_4_ and filtered to collect the organic phase, followed by evaporation of DCM under vacuum.

### POEGMA Synthesis and Purification

POEGMA was synthesized using ARGET‐ATRP, as described previously with minor changes.^[^ ^21b^ ^]^ Triethylene glycol methyl ether methacrylate (EG3) was passed through an alumina column to remove the polymerization inhibitor. The inhibitor‐free EG3 (10 mmol), the polymerization initiator (0.04 mmol), methanol (5.8 mL), and 100 mm NaCl (11.6 mL) were mixed in a flask (polymerization flask). A catalytic complex was prepared by mixing eight molar equivalents of tris(2‐pyridylmethyl) amine (TPMA) with the molar equivalent of copper II bromide (CuBr_2_) in water. The catalytic complex (0.08 mmol TPMA; 0.01 CuBr_2_) was transferred into the polymerization flask. The final volume was adjusted to 20 mL. In a separate flask, 64 mm ascorbic acid was prepared. Both flasks were purged with argon to remove oxygen. The polymerization was started with the addition of ascorbic acid into the polymerization flask at a rate of 1 µL min^−1^. The reaction was continued for 90 min. The resulting mixture was purified by passing it through an alumina column, followed by diethyl ether precipitation and evaporation under vacuum, yielding pure OH‐POEGMA.

### POEGMA Chain‐End Nitrophenyl Carbonate Activation

OH‐POEGMA (0.11 mmol) was dissolved in 5 mL anhydrous acetonitrile in a reaction flask (flask 1). In another flask (flask 2), bis(4‐nitrophenyl) carbonate (2.15 mmol) was dissolved in 15 mL anhydrous acetonitrile and transferred into flask 1. Finally, pyridine (5.16 mmol) was transferred to flask 1, and the final volume was adjusted to 21.5 mL using anhydrous acetonitrile. The resulting mixture was reacted for 16 h at room temperature. The precipitate was removed using glass fiber GF/B Filters (Whatman), followed by evaporating acetonitrile under vacuum until 0.5 mL of liquid remained. The resulting polymer mixture was purified by diethyl ether precipitation and was kept under vacuum to remove solvents. The NPC‐POEGMA was stored at −20 °C under an inert atmosphere.

### Structural Characterization of POEGMA

The chemical structure of the polymerization initiator and POEGMAs were analyzed by NMR spectroscopy. The compounds were dissolved in deuterated chloroform, and hydrogen NMR spectra were recorded using a 400 MHz Varian INOVA spectrometer. The data was analyzed using ACD/NMR software (ACD Labs). The NPC activation degree was defined as the mole % NPC present in POEGMA and was calculated as described in the Supporting Information.

### Conjugate Synthesis and Purification

NPC‐POEGMA or NPC‐PEG was weighed in flasks, followed by adding uricase into the flasks at a final concentration of 5 mg mL^−1^. The flasks were placed onto a rotator for 1 h at room temperature, allowing uricase to react with NPC‐functional polymers. A 100‐fold molar excess of POEGMA relative to the uricase tetramer was used for the synthesis of the uricase‐POEGMA conjugates. The uricase‐PEG_Rh_ and uricase‐PEG_Mw_ conjugates required 10 and 100 molar equivalents of NPC‐PEG (Creative PEGworks, NC). The resulting conjugates were purified by a single round of SEC on an AKTA Purifier equipped with a UV–vis detector operating at 220 and 280 nm and a HiLoad 26/600 Superdex 200 pg column (GE Healthcare) that was operated at a flow rate of 2 mL min^−1^ in a mobile phase of 10 mm carbonate, 100 mm NaCl, pH 9 buffer. The purified conjugates were buffer exchanged into 10 mm borate, pH 9 buffer using desalting columns with a 40 kDa MWCO (Pierce), followed by storage at −80 °C.

### Physical Characterization

Uricase variants were characterized for *M*_n_, *M*_w_, and *Ð* by SEC‐MALS using an Agilent 1260 HPLC equipped with a DAWN HELEOS II MALS detector (Wyatt Technology), an Optilab T‐rEX refractive index detector (Wyatt Technology), and a UV–vis detector operating at 280 nm (Agilent). The MALS detector was normalized using bovine serum albumin before each use. The mobile phase was 10 mm carbonate buffer with 150 mm NaCl and 100 ppm NaN_3_ (pH 10.3). The uricase variants were solubilized in the mobile phase, followed by filtration using 100 nm syringe filters (Whatman). 50 µL of the filtered solutions were separated on a WTC‐015N5 SEC column (5 µm; 150 Å; 4.6 mm internal diameter; Wyatt Technology). The flow rate was 0.5 mL min^−1^. The light scattering data were analyzed for *M*_n_, *M*_w_, *Ð*, and conjugation stoichiometry using ASTRA software (Wyatt Technology). The refractive index increment (d*n*/d*c*) and UV extinction coefficient were determined by the refractive index and UV–vis detectors, respectively. OH‐ and NPC‐POEGMA was characterized for *M*_n_, *M*_w_, and *Ð*, as described previously.^[^ ^21b^ ^]^

### Hydrodynamic Size Characterization

The *R*_h_ was characterized by DLS using a DynaPro Plate Reader (Wyatt Technology). Uricase and its polymer conjugates were buffer exchanged into PBS at pH 7.4 using Zeba desalting columns with a 40 kDa MWCO (Pierce), followed by filtration through a 100 nm syringe filter (Whatman). The measurements were recorded at 15 °C. Data were analyzed by a regularization fit for Raleigh spheres using Dynamics software (Wyatt Technology). In the stability experiment, uricase was cleaved from the ELP and buffer exchanged into PBS at pH 7.4 using a Zeba desalting column (Pierce), followed by measuring the DLS data at the specified time points.

### Biochemical Activity Assay

The activity of uricase and its polymer conjugates were quantified using an Amplex Red Uric Acid/Uricase Assay Kit (Thermo Scientific). In this assay, uricase catalyzed the conversion of uric acid to allantoin, hydrogen peroxide, and carbon dioxide. In the presence of horseradish peroxidase (HRP), hydrogen peroxide reacted stoichiometrically with the Amplex Red reagent to generate a red dye, which could be spectroscopically monitored kinetically. Serial dilutions of the uricase variants were prepared and tested by this assay. Following the manufacturer's protocols, the uric acid conversion activity (U) per mg uricase in each variant was reported.

### Endotoxin Purification

The conjugates were endotoxin purified using high‐capacity endotoxin removal columns (Pierce) according to the manufacturer's protocols, with minor changes. The mobile phase was 10 mm ammonium bicarbonate, pH 7.5, dissolved in endotoxin‐free water (Hyclone). The endotoxin‐purified conjugates were lyophilized and stored at −80 °C. The endotoxin content was tested using an Endosafe nexgen‐PTS instrument and cartridges (Charles River). The maximum acceptable endotoxin limit was 0.2 EU/kg mouse body weight. The samples were sterilized before administration into mice using a 0.22 µm Acrodisc syringe filter (Pall Corporation).

### Animal Experiments

The Duke Institutional Animal Care and Use Committee (IACUC) approved the in vivo studies described below. 6‐week‐old male C57BL/6J (Jackson Laboratories; stock no. 000664) were used for the in vivo studies. Mice were allowed to acclimate to the facilities for 1 week before starting the experiments. Mice were group‐housed in a photo‐controlled environment with 12 h dark/light cycles and were kept on a standard rodent diet with ad libitum access to water and food.

### Pharmacokinetics

The sterile and endotoxin‐free uricase variants were labeled with a fluorophore to track the drug in vivo.^[^ ^54^ ^]^ The lack of free dye was confirmed by HPLC equipped with a fluorescence detector. The uricase variants were administered i.v. into C57BL/6J mice (*n* = 4–5 mice per group) at a dose of 36.6 nmol kg^−1^. This dose value was determined by normalizing the recommended dose of pegloticase in humans (8 mg per injection) to the body surface area of mice using allometric scaling.^[^ ^55^ ^]^ Blood samples (10 µL) were collected from a small nick on the tail vein to track plasma concentrations of the treatments into heparin‐containing tubes (90 µL; 1000 U mL^−1^). Time points for the blood sampling were predose (−15 min), 40 s, 5, 30 min, 1, 2, 4, 8, and 24 h, and thereafter every 24 h up to 144 h. The samples were processed for plasma by centrifugation at 1600 × *g* for 15 min. The fluorophore concentration in the plasma samples was measured using a Victor plate reader (PerkinElmer) at 485 nm (excitation) and 535 nm (emission) (*n* = 3 wells per mouse). The plasma concentration of the drugs was plotted as a function of sampling time, followed by fitting to a one‐phase decay curve using GraphPad Prism 9 software. The PK parameters were identified using a noncompartmental PK analysis. The drug concentration was approximated to the concentration at *t* = 0 (*C*_o_)—termed initial drug concentration—and calculated from the intersection of the fitted decay curve with the *y*‐axis at *t* = 0. The elimination rate (*k*) was calculated from the slope of a linear regression curve fitted to the logarithm of drug concentration as a function of sampling time. The elimination half‐life was defined as the time needed for the drug concentration to reach its half‐maximal value and calculated using *k*. The effect of the ADAs on the drug PK was tested in a repeated PK assay as described above. The mice were weekly administrated with the same drugs a total of five times. Drug PK was tracked after the first injection and the fifth injection.

### Immunogenicity

Sterile and endotoxin‐free uricase‐POEGMA and uricase‐PEG_Mw_ were administered s.c. into separate cohorts of C57BL/6J mice (*n* = 10) every 17 days three times at a dose of 36.6 nmol kg^−1^ of conjugate equivalent using the same injection volume of endotoxin‐free PBS as a negative control. Next, ≈180 µL of blood were collected from the submandibular vein 10 days after each injection into lyophilized heparin‐containing tubes and processed into plasma, as described above. The plasma samples were stored at −80 °C until ADA analysis.

### Analysis of Anti‐Drug Antibodies

ADAs were analyzed using a previously described Luminex multiplexed assay^[^ ^21b^ ^]^ with minor modifications. Briefly, the plasma samples were diluted 500‐fold in PBS. Diluted plasma samples (50 µL) were transferred to a black 96‐well‐plate (Corning). Next, OVA‐, OVA‐PEG‐, and OVA‐POEGMA‐coupled magnetic beads were added to each well (50 µL; 2500 beads per set) and incubated for 1 h on a plate shaker. The magnetic beads were separated on a magnetic ring, and wells were washed with 0.2% w/v I‐Block (Thermo Scientific) in PBS (Hyclone)—termed the assay buffer. To analyze IgGs, R‐Phycoerythrin‐conjugated goat anti‐mouse IgG (Jackson Immunoresearch; #115‐115‐164) was transferred to the wells (5 µg mL^−1^; 100 µL) and incubated for 1 h. To analyze IgMs, biotin‐conjugated goat anti‐mouse IgM (Jackson Immunoresearch; #115‐065‐075) was used at the same concentration and volume and for the same duration. Next, the wells were washed with the assay buffer. For the analysis of IgM‐class ADAs, streptavidin‐R‐phycoerythrin (SAPE; Invitrogen) was transferred into the wells (7.5 µg mL^−1^; 100 µL) and incubated for 30 min, followed by washing the wells with the assay buffer. The beads were resuspended in 100 µL assay buffer, and their fluorescence signal was measured by MAGPIX (Luminex).

### Indirect and Competitive ELISA

The authors probed the reactivity of uricase, uricase‐PEG_Mw_, and uricase‐POEGMA toward PEG antibodies present in a murine plasma sample. A PEG antibody‐positive murine plasma sample was available from a previous study, where C57BL/6J mice (*n* = 10) were repeatedly exposed to OVA‐PEG emulsified in Freund's adjuvant, followed by blood collection and processing into plasma.^[^ ^21b^ ^]^ The plasma samples collected from each mouse at the end of the study (Day 44) were mixed at equal volumes, resulting in a PEG‐antibody‐positive plasma pool.

In indirect ELISA, first, 100 µL of OVA‐PEG, uricase‐PEG_Mw_, and uricase‐POEGMA solutions in carbonate buffer pH 9.2 were coated on 96‐well‐plate surface, such that there was 5 µg of PEG or POEGMA in each well. The uricase‐coated wells matched with the conjugate‐coated wells in the amount of uricase in each well. The antigen solutions were incubated overnight at 4 °C. The wells were blocked with 1% w/v iBlock (Thermo Scientific) in PBS (Hyclone)—termed blocking buffer—for an hour, followed by PBS‐washing twice. Next, the pooled PEG antibody‐positive murine plasma sample was diluted 500‐fold in PBS, and 100 µL of the diluted plasma sample was transferred to each well, followed by incubation for an hour and PBS‐washing twice. A biotinylated goat anti‐mouse IgM antibody (Jackson Immunoresearch; 115‐065‐075) was diluted in PBS (50 ng mL^−1^), and 100 µL of the resulting solution was transferred to each well. The antibody solution was incubated for an hour and removed from the wells, followed by PBS‐washing twice. Streptavidin‐poly HRP (Pierce) was diluted in PBS to 0.1 µg mL^−1^ and transferred to the wells, followed by a 30 min incubation. The excess solution was removed, and the wells were washed with PBS. TMB substrate (Pierce) was added to wells (50 µL) and incubated for 10 min, followed by being stopped with the addition of 2N sulfuric acid (50 µL) and absorbance reading on a plate reader (Eppendorf) at 450 nm. The absorbance was measured using a Victor plate reader (PerkinElmer).

In competitive ELISA, uricase, uricase‐PEG_Mw_, and uricase‐POEGMA competed with an unrelated PEGylated drug (exendin‐PEG) for binding to the pooled PEG‐antibody‐positive murine plasma sample. Exendin‐PEG was a stoichiometric conjugate of PEG with an *M*_w_ of 10.2 kDa and was available from a previous study.^[^ ^21b^ ^]^ Exendin‐PEG was coated on the surface of 96‐well‐plates to yield 5 µg of PEG per well by overnight incubation at 4 °C. The murine plasma sample was diluted 250‐fold in PBS. Uricase, uricase‐PEG_Mw_, and uricase‐POEGMA were prepared at varying concentrations up to 40 µm. 250 µL of the resulting solutions were mixed with 250 µL of the diluted plasma, followed by overnight incubation on a rotator at 4 °C. On the day of the assay, exendin‐PEG was removed from the wells, and the wells were blocked with the blocking buffer for 1 h at room temperature, followed by PBS‐washing twice. 100 µL of the resulting antigen:plasma mixtures were transferred to each well and incubated for 1 h. The drugs were removed from the wells, followed by PBS‐washing twice. Next, a biotinylated goat anti‐mouse IgM antibody (Jackson Immunoresearch; 115‐065‐075) was diluted in PBS (50 ng mL^−1^), and 100 µL of the resulting solution was transferred to each well. The antibody solution was incubated for 1 h and removed from the wells, followed by PBS‐washing twice. Streptavidin‐poly HRP (Pierce) was diluted in PBS to 0.1 µg mL^−1^ and transferred to the wells, followed by a 30 min incubation. The excess solution was removed, and the wells were washed with PBS. TMB substrate (Pierce) was added to wells (50 µL) and incubated for 10 min, followed by being stopped with the addition of 2 n sulfuric acid (50 µL) and absorbance reading on a plate reader (Eppendorf) at 450 nm. The absorbance was measured using a Victor plate reader (PerkinElmer).

### Statistical Analyses

For all studies, except for in vivo experiments, each treatment was performed in triplicate and the mean values of all were computed with no exception for outliers. Furthermore, each experiment was performed on different days to enable statistical analysis and ensure the reproducibility of results. Mice were randomly distributed into groups. The sample size was estimated using open‐source G‐power software based on literature studies and the authors' previous experience and was increased from initial estimates when necessary to achieve enough statistical power to detect differences among groups. The experiments that did not involve animals were independently replicated at least twice and repeated at least three times. The PK experiments used at least *n* = 3 mice per group, while *n* = 10 mice per treatment were used in the immunogenicity experiments. The data represented the mean response of the treatment group ± standard error of the mean, unless otherwise noted. The data were analyzed using two‐way ANOVA, followed by post‐hoc Tukey's multiple comparison tests. *p* < 0.05 indicated a statistically significant test. (*p < 0.05; **p < 0.01; ***p < 0.001; ****p < 0.0001). Not significant (ns). GraphPad Prism 9.0 was used for all statistical analyses.

## Conflict of Interest

I.O. and A.C. have a pending US patent application on nonimmunogenic PEG‐like polymer conjugate technology (U.S. 63/169,541). I.O., A.M.H., and A.C. have a pending patent application on the uricase conjugates of POEGMA. I.O., A.M.H., and A.C. have equity in Gateway Bio, a company that is commercializing the POEGMA conjugate technology.

## Author Contributions

I.O., A.M.H., and A.C. conceived the study. I.O. and A.C. designed the experiments and wrote the manuscript. I.O., G.K., R.G., X.L., N.Z., and P.S. performed the experiments. S.K.N., J.H.C., and M.S.H. provided expertise with experimental design and data interpretation. Data were analyzed by I.O., G.K., R.G., X.L., N.Z., and A.C. All authors contributed to the interpretation of the data.

## Supporting information

Supporting Information

## Data Availability

The data that support the findings of this study are available in the supplementary material of this article.
