# Supplementary material for: Polyethylene Glycol‐Like Brush Polymer Conjugate of a Protein Drug Does Not Induce an Antipolymer Immune Response and Has Enhanced Pharmacokinetics than Its Polyethylene Glycol Counterpart
Source: Adv Sci (Weinh). 2022 Feb 8;9(11):2103672. doi: 10.1002/advs.202103672 (PMC9008788; doi:10.1002/advs.202103672)
Supplement: Supplementary file 1 — Supporting Information [file ADVS-9-2103672-s001.pdf]

WILEY-VCH

ADVANCED SCIENCE
Open Access

Supporting Information

for *Adv. Sci.*, DOI: 10.1002/advs.202103672

PEG-like brush polymer conjugate of a protein drug has enhanced pharmacokinetics than a PEG conjugate and does not induce an anti-polymer immune response

*Imran Ozer, Garrett Kelly, Renpeng Gu, Xinghai Li, Nikita Zakharov, Parul Sirohi, Smita K. Nair, Joel H. Collier, Michael S. Hershfield, Angus M. Hucknall, and Ashutosh Chilkoti**

Supporting Information

PEG-like brush polymer conjugate of a protein drug has enhanced pharmacokinetics than a PEG conjugate and does not induce an anti-polymer immune response

Imran Ozer, Garrett Kelly, Renpeng Gu, Xinghai Li, Nikita Zakharov, Parul Sirohi, Smita K. Nair, Joel H. Collier, Michael S. Hershfield, Angus M. Hucknall, and Ashutosh Chilkoti[*]

Supplementary Figures

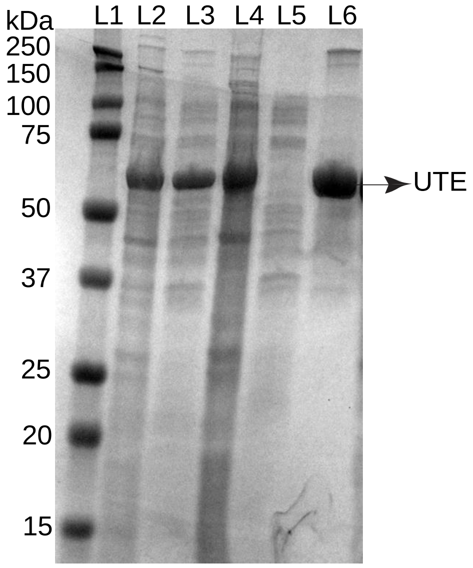

Figure S1. Coomassie-stained SDS-PAGE of UTE purification by ITC. *Candida utilis* uricase was recombinantly produced in *E. coli* with a C-terminal TEV protease cleavage site and an ELP expression tag. Cycles of alternating hot and cold centrifugation steps were used to purify the fusion protein by the reversible LCST phase transition behavior conferred by the ELP tag. Lane (L). L1: Ladder; L2: Lysate; L3: Insoluble fraction of lysate; L4: soluble fraction of lysate; L5: Hot spin supernatant round 1; L6: Cold spin supernatant round 1. This ITC cycle was repeated for one or two more cycles to obtain UTE with a purity > 95%, as quantified by densitometry of SDS-PAGE gels.

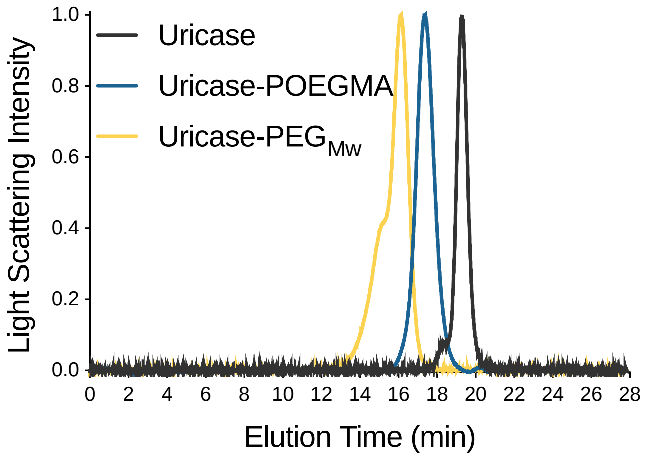

Figure S2. SEC-MALS traces of uricase variants. Uricase comprised 0.8 mass % octamer, corresponding to the peak eluting between 18-19 min while the major peak eluting at 19-21 min corresponds to the uricase tetramer.

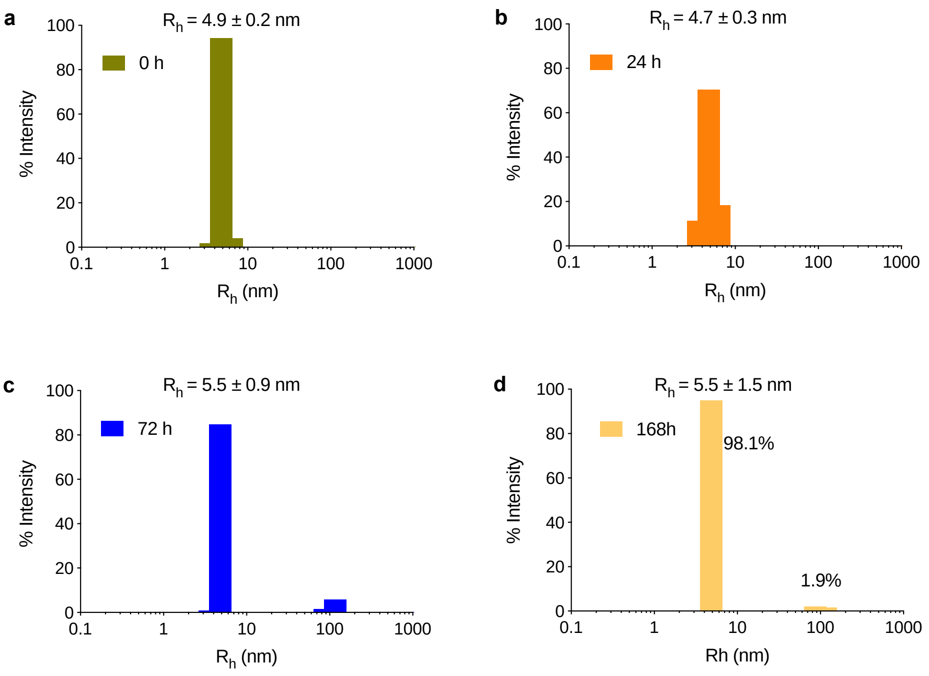

Figure S3. Stability of uricase. The R_h of uricase tetramer **(A)** immediately after ELP cleavage and at **(B)** 24 h, **(C)** 72 h, and **(D)** 168 h, as measured by DLS. The uricase tetramer contained only 1.9 mass% aggregates at 168 h, indicating its high stability at physiological conditions.

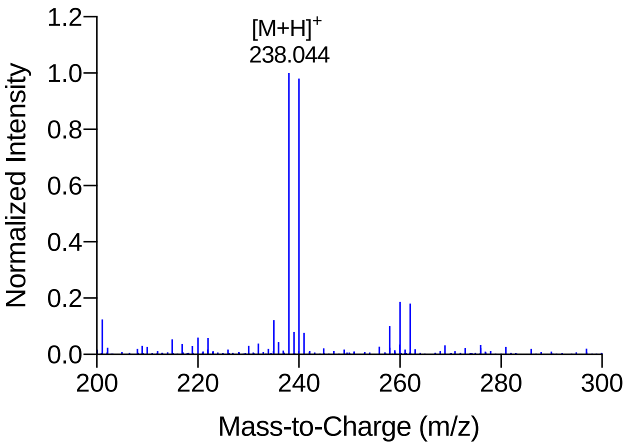

Figure S4. High resolution mass spectrum of the polymerization initiator. The theoretical mass [M] of the polymerization initiator ($C_8H_{16}BrNO_2$) was calculated as 237.04 Da using ChemDraw software, and the major peak at 238.044 Da was confirmed as the $[M+H]^+$ ion by high-resolution mass spectrometry (HRMS). The peak at 240.044 Da is due to the isotopic distribution of bromine (78.918 Da; 50.7% and 80.918 Da; 49.3%).

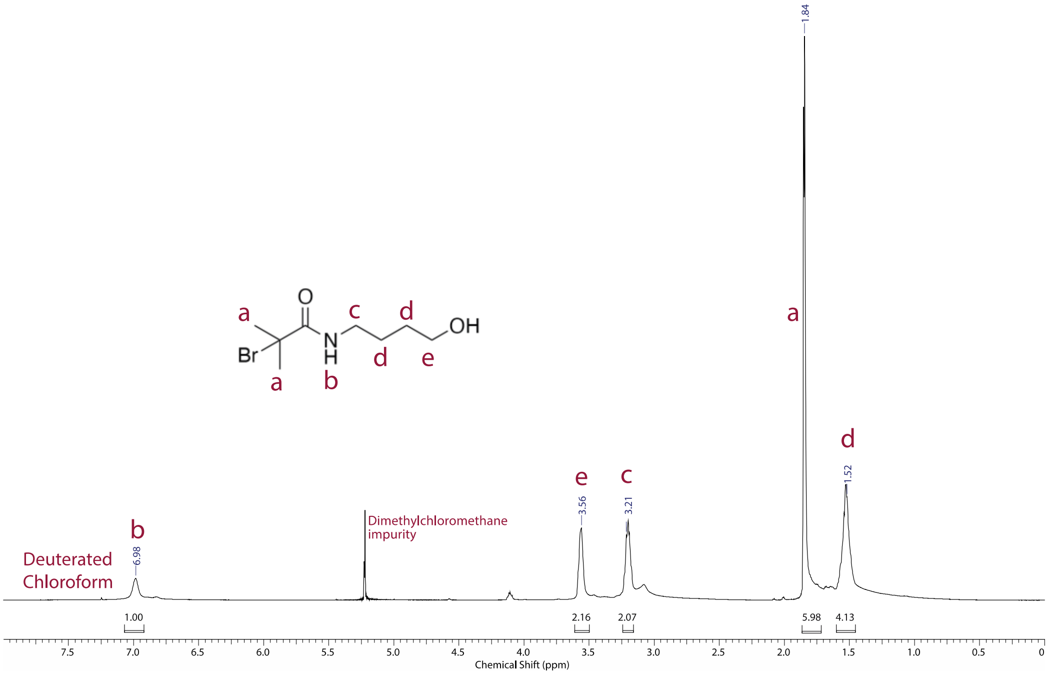

Figure S5. Structural analysis of the polymerization initiator. The initiator structure was analyzed by ^{1}H-NMR spectroscopy. Data were acquired on a 400 MHz Varian Inova spectrometer and analyzed by ACD/NMR software (ACD Labs). Deuterated chloroform was used as a solvent. Integration values show the average number of hydrogen atoms present. ^{1}H NMR (400 MHz, $CDCl_3$-d_6): 6.98 (b, 1H, NH), 3.56 (e, 2H; CH_2), 3.21 (c, 2H; CH_2), 1.84 (a, 6H; CH_3), 1.52 (d, 2H; CH_2).

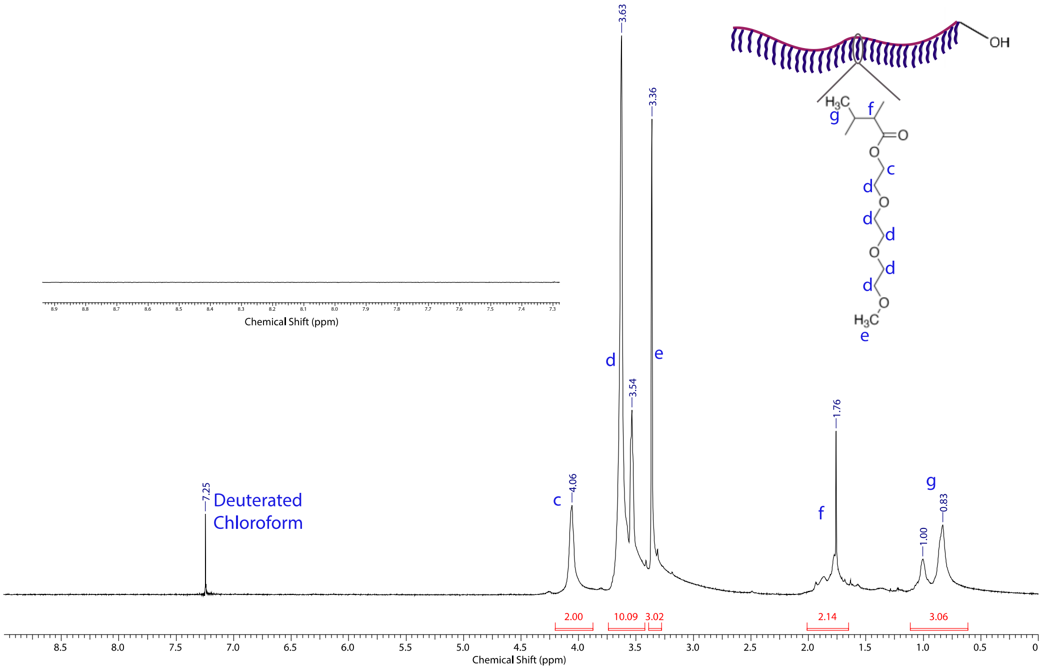

Figure S6. Structural analysis of OH-POEGMA. The OH-POEGMA structure was analyzed by H-NMR spectroscopy. Integration values showed the average number of hydrogen atoms present in methylene protons (c; 2H; 4.0-4.4 ppm), OEG side-chain (d; 10H; 3.4-4.4 ppm), OEG chain end-group (e; 3H; 3.3-3.5 ppm), backbone (f; 2H; 1.5-2.0 ppm), and backbone methacrylate (g; 3H; 0.6-1.2 ppm). Data were acquired on a 400 MHz Varian Inova spectrometer and analyzed by ACD/NMR software (ACD Labs). Deuterated chloroform was used as a solvent.

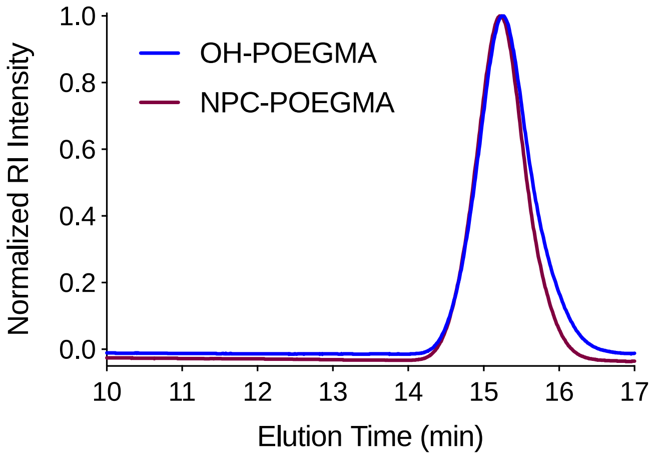

Figure S7. GPC traces of OH-POEGMA and NPC-POEGMA. The near-identical chromatograms show that NPC activation did not alter the POEGMA structure.

Table S1. Physical characterization of the polymers used to synthesize uricase conjugates. The M_n, M_w, and $Đ$ were determined from the GPC-MALS data shown in Figure S7 and analyzed by ASTRA software (Wyatt Technology). The R_h was measured by DLS in PBS at pH 7.2 (n=10).

Polymer	*Mn* (kDa)	M_w (kDa)	$Đ$	R_h (nm)
NPC-PEG	9.9	10.4	1.05	2.8 ± 0.6
OH-POEGMA	10.0	10.1	1.01	1.5 ± 0.5
NPC-POEGMA	9.7	10.3	1.06	1.6 ± 0.4

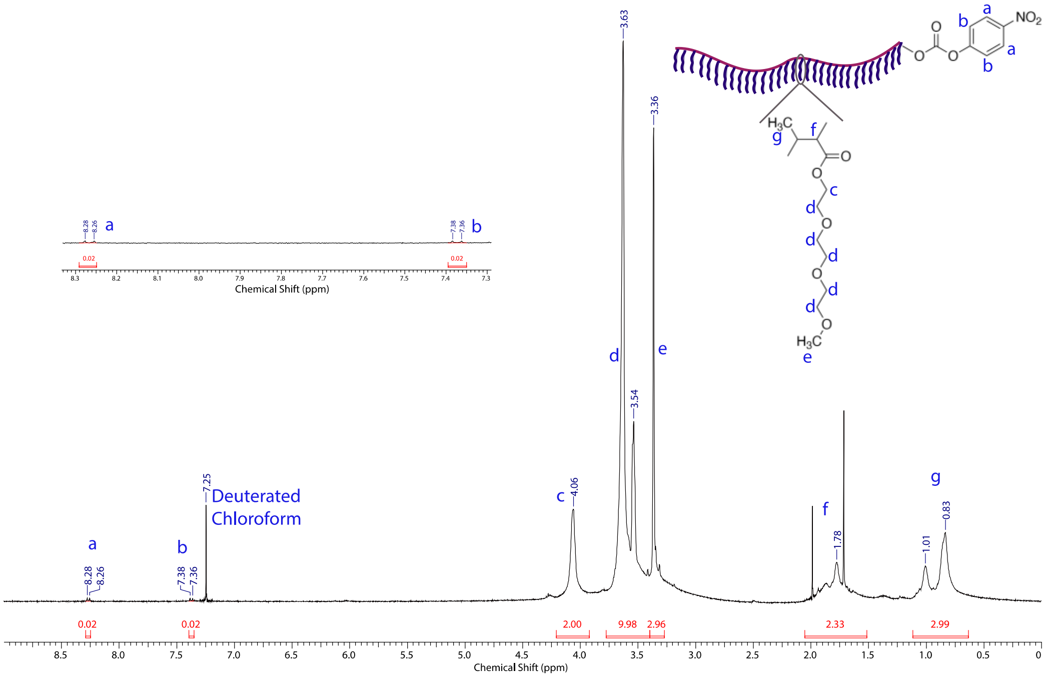

Figure S8. Structural analysis of NPC-POEGMA. The NPC-POEGMA structure was analyzed by NMR spectroscopy. Integration values show the average number of hydrogen atoms present in methylene protons (c; 2H; 3.8-4.2 ppm), OEG side-chain (d; 10H; 3.4-4.4 ppm), OEG chain end-group (e; 3H; 3.3-3.5 ppm), backbone (f; 2H; 1.5-2.0 ppm), and backbone methacrylate (g; 3H; 0.6-1.2 ppm). The presence of NPC end-group was confirmed by the benzene peaks (a; 1H; 8.26 ppm and b; 1H; 7.38 ppm). The proportion of the benzene peaks' total integration (a+b) to that of methylene protons (c) was used to calculate the NPC end-activation per EG3 monomeric unit, as described in Supplementary Methods. Data were acquired on a 400 MHz Varian Inova spectrometer and analyzed by ACD/NMR software (ACD Labs). Deuterated chloroform was used as a solvent.

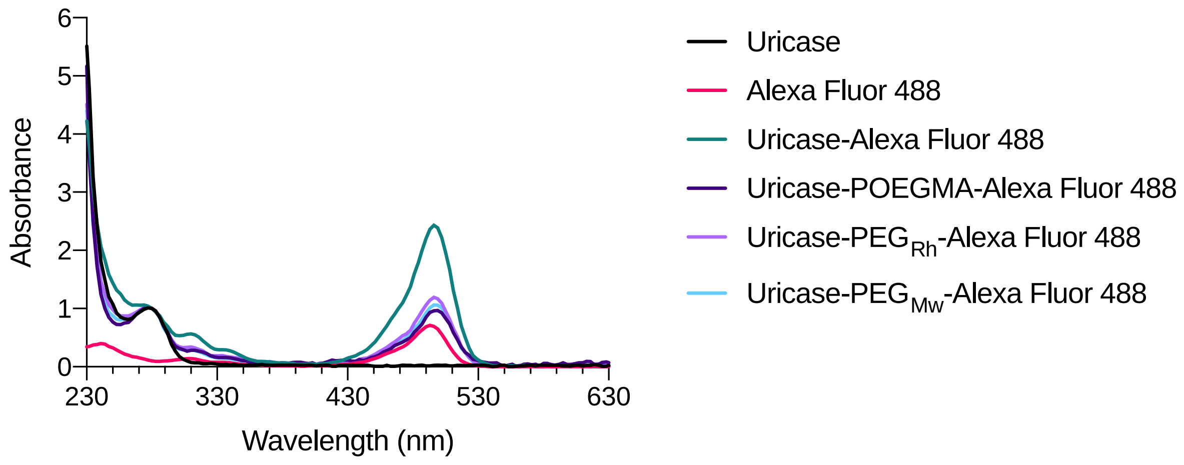

Figure S9. UV-vis spectrum of Alexa-Fluor 488 labeled uricase variants used to measure PK. UV-vis absorbance spectrum was determined on a Nanodrop spectrometer. Unmodified uricase and Alexa Fluor 488 were used as controls.

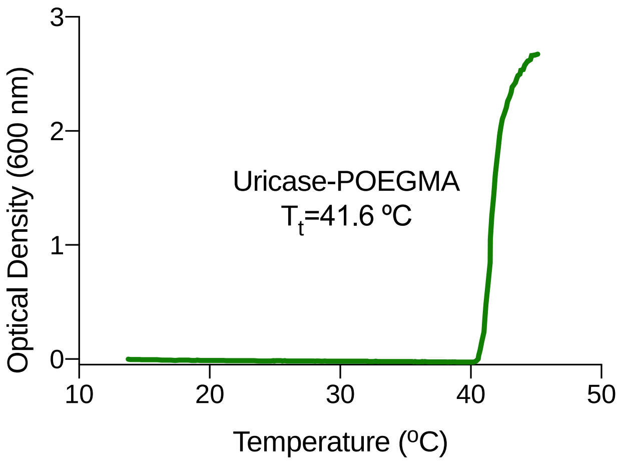

Figure S10. The LCST phase behavior of uricase-POEGMA. The optical density was measured at 600 nm on a temperature-controlled UV-vis spectrometer (Cary 300 Bio; Varian) in PBS. The temperature was increased at a rate of 1°C per minute. The sharp increase in the optical density indicates the phase transition. The transition temperature (T_t) was defined as the maximum of the first derivative of the curve.

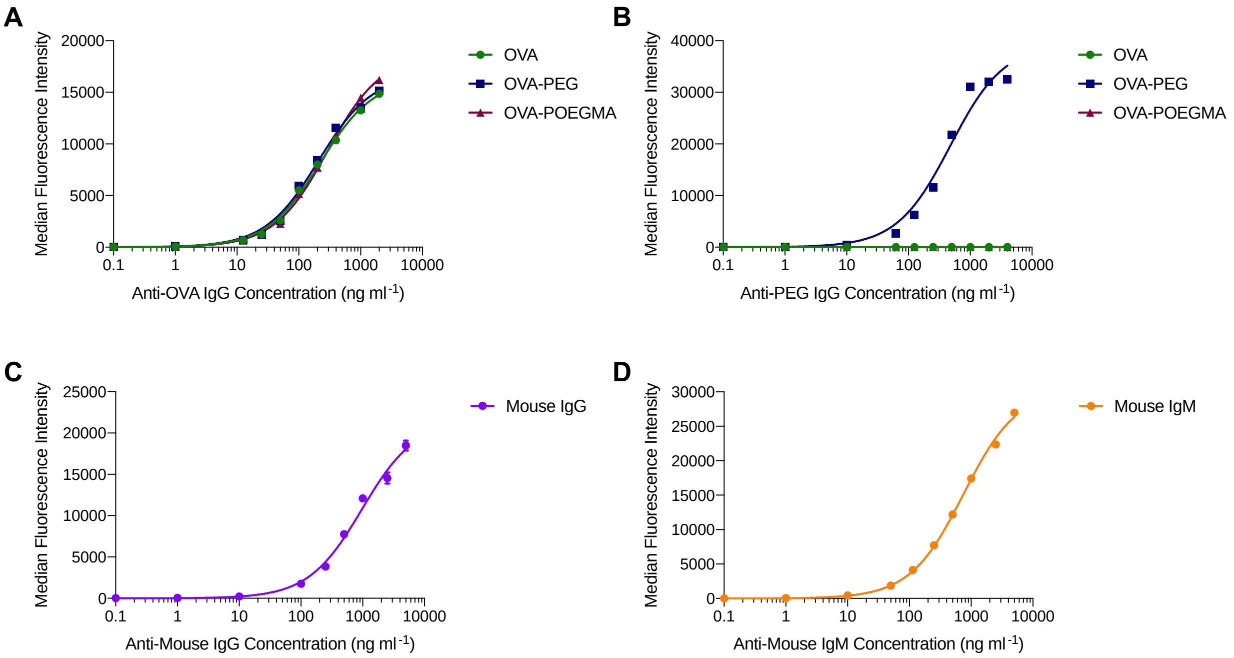

Figure S11. Characterization of drug- and antibody-coupled beads used in Luminex multiplexed immunoassays. The amount of drug coupled was carefully titrated to ensure that the resulting bead sets had equal amounts of the same type of antigen, indicated by near-identical MFI detected at varied positive control antibody concentrations. The data given here was published in reference.[22b]

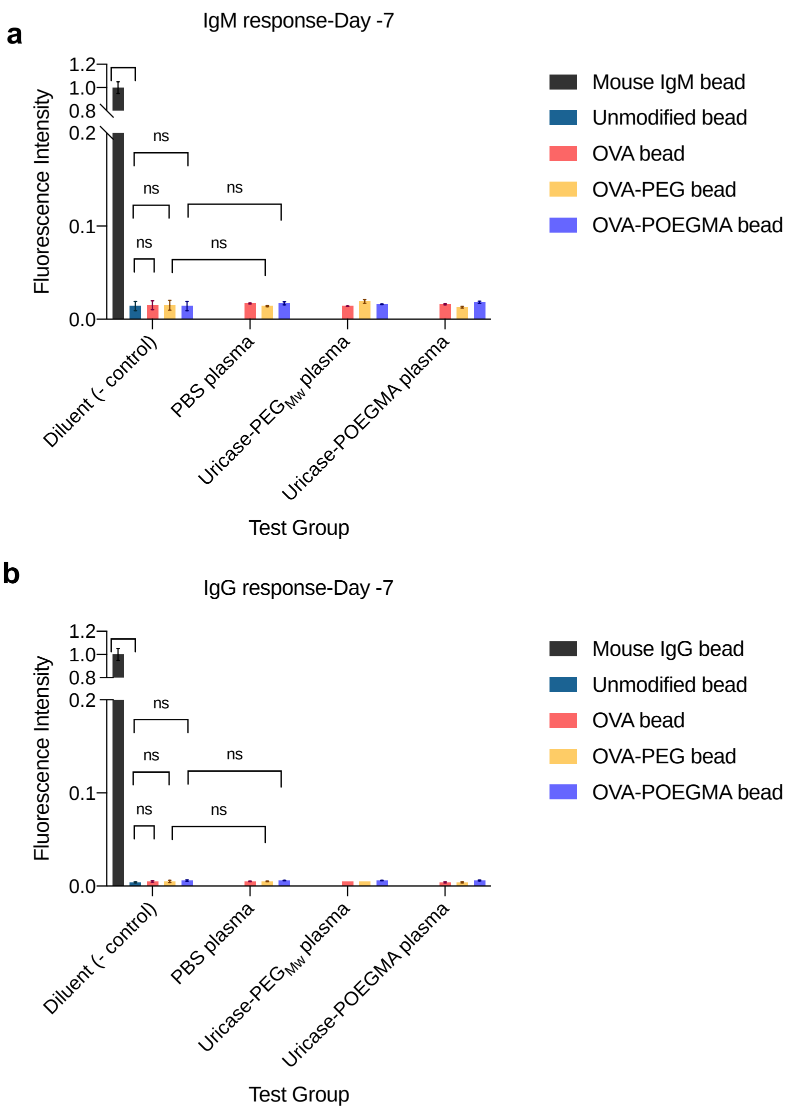

Figure S12. The pre-dose ADA response of mice treated in the immunogenicity experiment. (A) IgM and **(B)** IgG response 7 days before the first administration of the treatments (n=10 mice per group). ADA response was measured for each mouse using a Luminex multiplexed assay (n=6). OVA-PEG- and OVA-POEGMA-coupled beads show the PEG-specific and POEGMA-specific immune response in plasma samples of mice treated with PBS, uricase-PEG_{Mw} and uricase-POEGMA. Data were normalized to the signal measured with mouse IgG- and IgM-coupled beads (positive controls) and are plotted as the average ADA response in a treatment group ± SEM. Data were analyzed by two-way ANOVA, followed by *post-hoc* Tukey's multiple comparison test. A test was considered statistically significant when p<0.05. * p<0.05, **p<0.01, *** p<0.001, and ****p<0.0001. Not significant (ns).

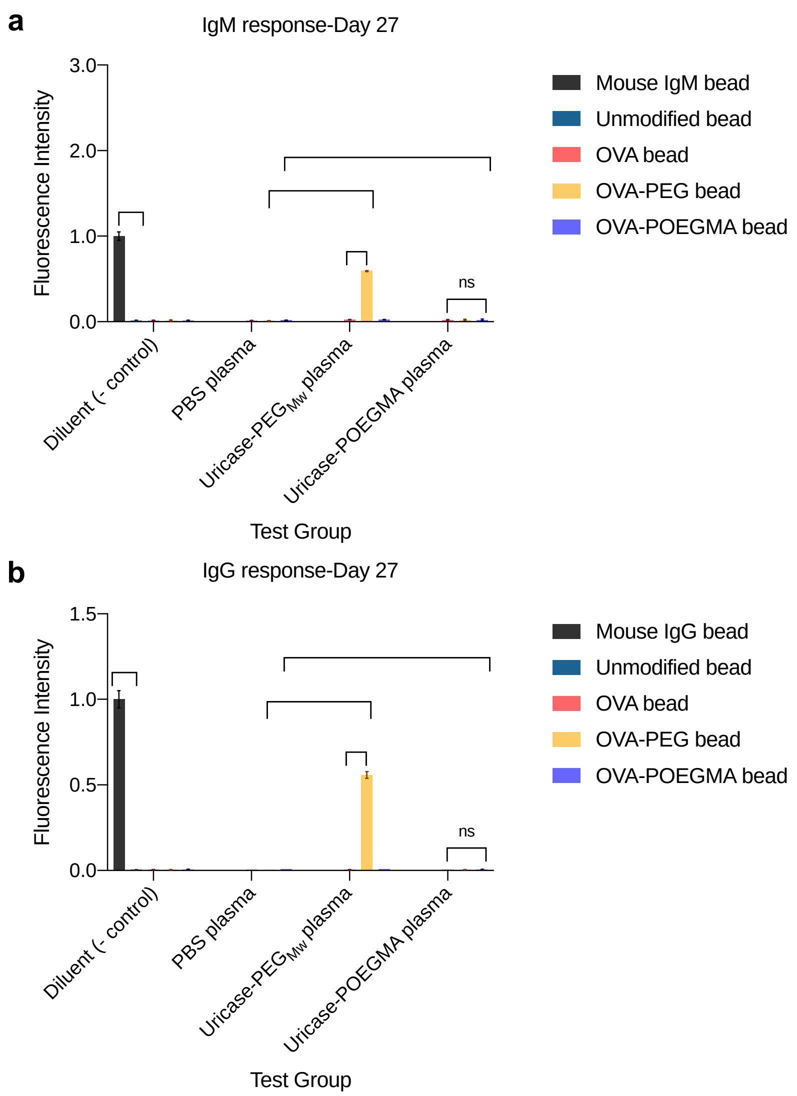

Figure S13. The ADA response on Day 27. (A) IgM and **(B)** IgG response on Day 27 (n=10 mice per group). ADA response was measured for each mouse using a Luminex multiplexed assay (n=6). OVA-PEG- and OVA-POEGMA-coupled beads show the PEG-specific and POEGMA-specific immune response in plasma samples of mice treated with PBS, uricase-PEG_{Mw} and uricase-POEGMA. Data were normalized to the signal measured with mouse IgG- and IgM-coupled beads (positive controls) and are plotted as the average ADA response in a treatment group ± SEM. Data were analyzed by two-way ANOVA, followed by *post-hoc* Tukey's multiple comparison test. A test was considered statistically significant when $p<0.05$. * $p<0.05$, **$p<0.01$, *** $p<0.001$, and ****$p<0.0001$. Not significant (ns).

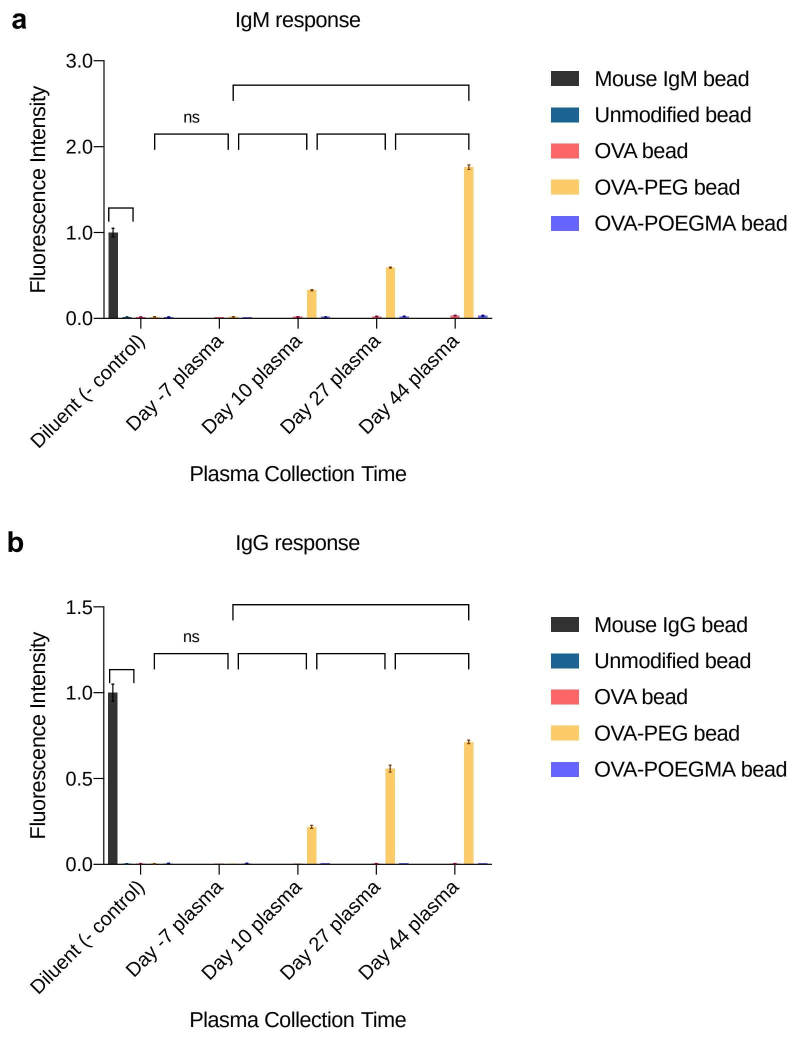

Figure S14. The development of the ADA response over time. (A) IgM and **(B)** IgG response (*n*=10 mice per group). ADA response was measured for each mouse using a Luminex multiplexed assay (*n*=6). OVA-PEG- and OVA-POEGMA-coupled beads show the PEG-specific and POEGMA-specific immune response in plasma samples of mice treated with PBS, uricase-PEG_{Mw} and uricase-POEGMA. Data were normalized to the signal measured with mouse IgG- and IgM-coupled beads (positive controls) and are plotted as the average ADA response in a treatment group ± SEM. Data were analyzed by two-way ANOVA, followed by *post-hoc* Tukey's multiple comparison test. A test was considered statistically significant when $p<0.05$. * $p<0.05$, **$p<0.01$, *** $p<0.001$, and ****$p<0.0001$. Not significant (ns).

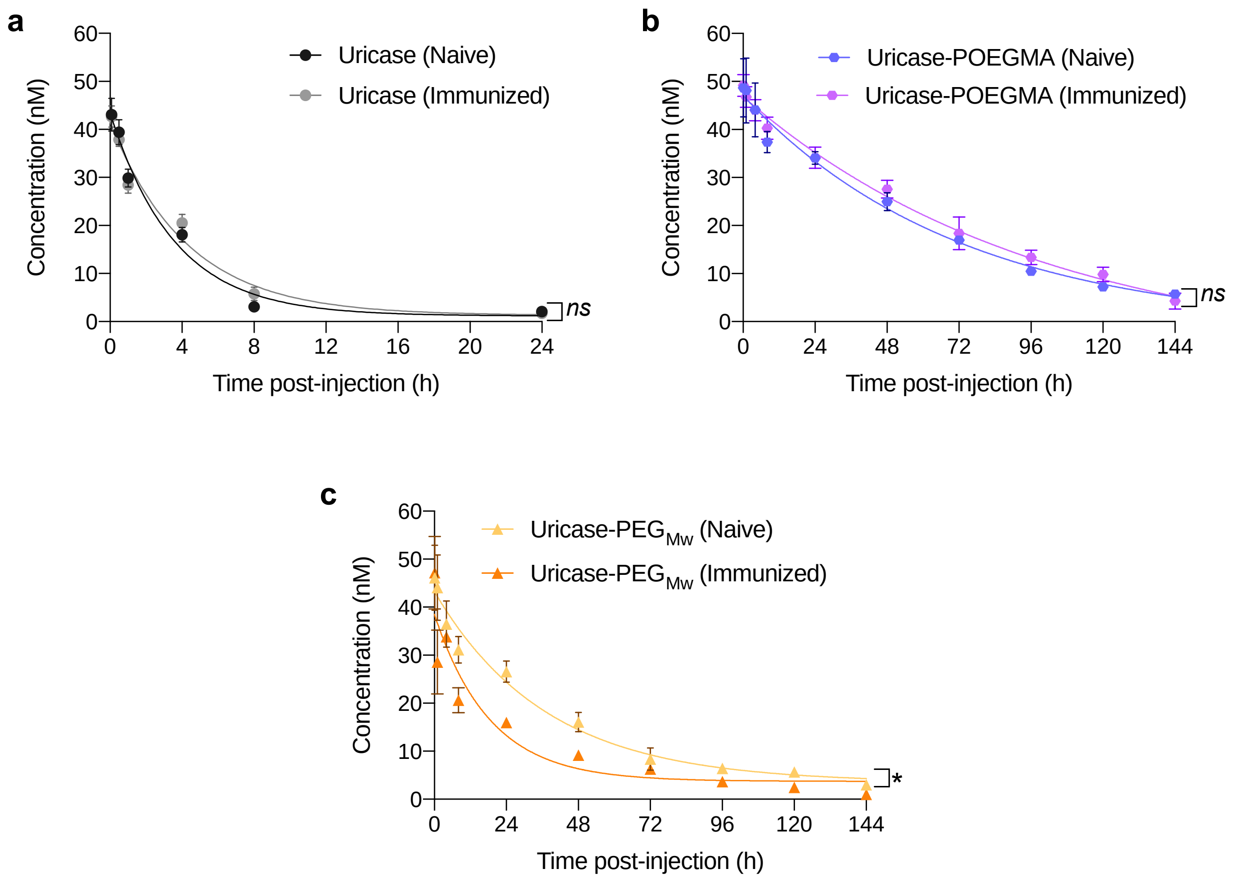

Figure S15. The effect of ADAs on drug PK. Plasma concentrations of sterile, endotoxin-free, and fluorescently labeled **(A)** unmodified uricase, **(B)** uricase-POEGMA, and **(C)** uricase-PEG_{Mw} after the first (naïve) and fifth (immunized) *i.v.* administration to C57BL/6J mice (*n*=5) at 36.6 nmol kg^{-1} dose of conjugate equivalent. Plasma drug concentrations were tracked by collecting blood at pre-determined time points for 144 h, followed by processing them into plasma and measuring the plasma fluorescence on a fluorescence plate reader. Data were fit to a one-phase exponential decay curve using GraphPad Prism 9. PK parameters are shown in Table 2. The naïve mice data are also shown in Figure 3.

Supplementary Methods

NPC activation degree calculation: NPC activation degree was calculated using Equation 1, where the proportion of the hydrogens present in the benzene ring of NPC (Peak a (2H) at 8.26 ppm; Peak B (2H) at 7.38 pm) to the methylene protons (2H; 4.0-4.4 ppm) resulted in the NPC end-activation degree per EG3 monomeric unit. The activation degree per POEGMA chain was found by multiplying the NPC end-activation degree per EG3 monomeric unit with the degree of polymerization (DP) of POEGMA. The DP is the number of EG3 chains present in a POEGMA chain and was calculated by dividing POEGMA M_w by M_w of EG3 monomer.

$$\% \, NPC \ activation = \frac{\left(\int_{8.25ppm}^{8.29ppm} peak\ a + \int_{7.35ppm}^{7.39ppm} peak\ b\right) / 4}{\left(\int_{3.8ppm}^{4.2ppm} peak\ c\right) / 2} * DP * 100 \text{ (Equation 1)}$$
